# Effects of celery (*Apium graveolens*) on blood pressure, glycemic and lipid profile in adults: a systematic review and meta-analysis of randomized controlled trials

**DOI:** 10.3389/fnut.2025.1597680

**Published:** 2025-07-22

**Authors:** Dangzhen Liu, Haiyan Zhao, Hong Xu, Jingqing Hu

**Affiliations:** ^1^School of Basic Medical Sciences, Chengdu University of Traditional Chinese Medicine, Chengdu, China; ^2^School of Third Clinical Medical, Nanjing University of Traditional Chinese Medicine, Nanjing, China; ^3^School of Interdisciplinary Medicine, Tianjin University of Traditional Chinese Medicine, Tianjin, China; ^4^State Key Laboratory of Dampness Syndrome of Chinese Medicine, The Second Affiliated Hospital of Guangzhou University of Chinese Medicine, Guangzhou, China; ^5^XIN-Huangpu Joint Innovation Institute of Chinese Medicine, Guangzhou, China

**Keywords:** celery, meta-analysis, lipid profile, blood pressure, glycemic

## Abstract

**Background:**

Celery is commonly used as a diet intervention for hypertension, hyperglycemia, and hyperlipidemia. However, its precise therapeutic efficacy remains uncertain.

**Objective:**

This study aims to comprehensively evaluate the efficacy of celery preparations in regulating blood pressure, blood glucose, and blood lipids profiles in adults.

**Methods:**

A systematic search was conducted in PubMed, Web of Science, EMBASE, Scopus, Cochrane Library, Clinicaltrials.gov, China Biology Medicine disc, and China National Knowledge Infrastructure. Randomized controlled trials of celery were included. Data were analyzed using either a random-effects model or a fixed-effects model, depending on heterogeneity, and were presented as standardized mean differences (SMDs) with corresponding 95% confidence intervals (CIs). All eligible studies were evaluated in terms of study characteristics, risk of bias, me-ta-analysis, sensitivity analysis, meta-regression, and publication bias.

**Results:**

Our meta-analysis included ten randomized controlled studies with a total of 511 participants. The results demonstrated significant therapeutic effects of celery on systolic blood pressure (SMD: −1.0; 95% CI: −1.85 to −0.14), diastolic blood pressure (SMD: −0.93; 95% CI: −1.54 to −0.33), fasting plasma glucose (SMD: −0.80; 95% CI: −1.58 to −0.01), and triglyceride (SMD: −1.18; 95% CI: −1.45 to −0.91). However, no overall effects were observed on total cholesterol, low-density lipoprotein, or high-density lipo-protein. Subgroup analysis revealed that celery seeds or celery preparations exceeding 1,000 mg/day were more effective than other parts of celery. Additionally, no significant difference in adverse events between celery and placebo.

**Conclusion:**

This meta-analysis demonstrated that Celery preparations significantly improve hypertension, hyperglycemia, and hyperlipidemia, with a favorable safety profile. Celery seeds or celery preparations exceeding 1,000 mg/day will have better effect. These findings suggest that celery performs well as a potential dietary supplement for reducing hypertension, hyperglycemia, and hyperlipidemia. However, the substantial heterogeneity observed for most outcomes and limited sample sizes warrant further high-quality clinical trials with longer follow-up periods to confirm these effects and establish optimal dosing regimens.

**Systematic review registration:**

https://www.crd.york.ac.uk/PROSPERO/view/CRD42025631143, PROSPERO: CRD42025631143.

## 1 Introduction

Hypertension, hyperglycemia, and hyperlipidemia are interrelated conditions influenced by both genetic and environmental factors. Epidemiological data reveal alarming prevalence rates: hypertension affects approximately 1.28 billion individuals globally (1), with a prevalence of 40%−60% in developed countries (2). Hyperglycemia affects 9.1% of the global population (464 million people), and this figure is projected to rise to 10.0% (638 million) by 2045 (3). Hyperglycemia is often accompanied by lipid disorders (4) and acts synergistically with hypertension to elevate cardiovascular disease (CVD) risk. Compared with hypertension alone, their coexistence approximately doubles the risk of CVD (5), posing a significant burden on healthcare systems.

There is a growing interest in dietary interventions to complement conventional therapies and enhance their effectiveness. Celery, with its long-standing use in traditional medicine across various countries (6–8) and its unique phytochemical profile, has emerged as a promising candidate.

Apigenin, a bioactive compound in celery, exerts vasodilatory and antiproliferative effects on vascular smooth muscle cells, contributing to blood pressure reduction (69). Celery has also been demonstrated to effectively lower triglyceride and cholesterol levels in rat models (9, 10). Its flavonoids act as potent scavengers of reactive oxygen species, thereby reducing lipid peroxidation (11). Celery seed extract has been shown to regulate blood pressure through multiple mechanisms, including calcium channel blockade, β-adrenergic receptor inhibition, and diuretic activity (12).

3-n-butylphthalide, a compound present in celery seeds, has been found to improve insulin resistance and lower blood glucose levels (10). Both aqueous and ethanol extracts of celery seeds have shown lipid-lowering bioactivity in hamster models (13).

Several clinical studies support the above potential benefits. Clinical trials have also reported significant antihypertensive effects of celery (14, 15). For instance, a randomized trial conducted by Yusni et al. in prediabetic patients indicated that celery may reduce blood glucose levels (16). Additionally, randomized controlled trials have suggested that 1.34 g/day celery seeds contribute to lowering blood lipid levels (17).

Despite these promising findings, a comprehensive systematic review and meta-analysis of celery's effects on these parameters remains lacking. This study aims to systematically evaluate the evidence on the effects of celery on blood pressure, blood glucose, and lipid profiles, and to assess its safety profile to inform clinical practice.

## 2 Materials and methods

This study was conducted in accordance with the Preferred Reporting Items for Systematic Reviews and Meta-Analyses (PRISMA) guidelines (18) and has been registered in PROSPERO (Registration No.: CRD42025631143, https://www.crd.york.ac.uk/PROSPERO/display_record.php?RecordID=631143).

### 2.1 Search strategy

A systematic search was performed in PubMed, Web of Science, EMBASE, Scopus, Cochrane Library, ClinicalTrials.gov, China Biology Medicine disc, and the China National Knowledge Infrastructure from database inception to January 15, 2025. MeSH terms and free-text keywords were used as appropriate for each database. The detailed search strategy is provided in Supplementary material 1.

This study included publications in all languages. Two reviewers (D.L. and H.X.) independently conducted an initial screening of identified titles and abstracts to determine their eligibility, followed by a full-text review when necessary. To minimize the risk of missing relevant studies, reference lists of included articles and related reviews were manually checked. Study selection was performed independently by two reviewers (D.L. and H.X.) following the PICOS framework, and any discrepancies were resolved through consultation with a third reviewer (H.Z.). The PICOS criteria were defined as follows:

(1) Participants: Adults;
(2) Intervention: Celery;
(3) Control: Others;
(4) Outcomes: systolic blood pressure (SBP), diastolic blood pressure (DBP), fasting plasma glucose (FPG), triglycerides (TG), high-density lipoprotein cholesterol (HDL-c), low-density lipoprotein cholesterol (LDL-c), total cholesterol (TC), and safety information;
(5) Study design: Randomized controlled trials (RCTs).

### 2.2 Eligibility criteria

Inclusion Criteria: (1) Celery was used as the intervention. (2) A non-celery intervention was used as the control. (3) The study was a RCT. (4) Participants were adults (18 years old and above).

Exclusion Criteria: (1) Incomplete data. (2) Animal or cell-based experiments, study protocols, case reports. (3) Reviews, commentaries, conference papers or letters to the editor.

### 2.3 Data extraction and risk of bias assessment

Following the guidelines of the Cochrane Handbook for Systematic Reviews of Interventions (19), the following data were extracted:

(1) Study characteristics: first author, publication year, country, population, sample size, study design, and type of control group;
(2) Participant characteristics: mean age and sex distribution;
(3) Intervention and comparator details: intervention method, dosage, and duration;
(4) Outcome measurements: pre- and post-treatment mean and standard deviation (SD) for SBP, DBP, FPG, TC, HDL-C, LDL-C, and TG, or the mean difference and SD between pre- and post-treatment values.

The quality of included studies was assessed using the Risk of Bias 2 (RoB 2) tool recommended by the Cochrane Collaboration (20). The following domains were evaluated: randomization process, deviations from intended interventions, missing outcome data, measurement of the outcome, selection of the reported result, and overall bias. The certainty of evidence used the GRADE (Grading of Recommendations Assessment, Development and Evaluation) approach (21); detailed criteria are provided in Supplementary material 3.1.

### 2.4 Data synthesis and analysis

All statistical analyses were performed using STATA 17.0. Meta-analyses were conducted using post-treatment means and SDs when baseline characteristics showed no significant differences. If baseline differences were significant or not reported, the mean change and the SD of change were used instead. According to the Cochrane Handbook (22, 23), when the SD of change scores was not reported, it was calculated using the formula: $SD_{change}=\sqrt{SD_{baseline}^{2}+SD_{final\;}^{2}-2\cdot r\cdot SD_{baseline}\cdot SD_{final}}$. The *r* value is calculated using the following formula: $r=\frac{SD_{baseline}^{2}+SD_{final\;}^{2}-SD_{change\;}^{2}}{2\cdot SD_{baseline}\cdot SD_{final}}$. The estimated *r* values were 0.8 for statistically significant pre-post differences and 0.356618 for non-significant differences (24). For crossover trials with two treatment periods and a washout phase, only the first period (i.e., prior to the onset of the washout phase) data are included in the analysis. This approach is adopted to minimize the potential influence of carryover effects on the results.

Standardized mean differences (SMDs) with 95% confidence intervals (CIs) were used to assess treatment effects. Heterogeneity was evaluated using the I^2^ statistic, where an I^2^ value >50% indicated substantial heterogeneity (25). A random-effects model was applied when I^2^ >50%, whereas a fixed-effects model was used when I^2^ ≤ 50%. For subgroup analysis [conducted when at least six studies were available (26)], potential sources of heterogeneity and factors affecting the effect of celery treatment were explored based on type of control (pharmacological vs. other interventions), intervention duration (≥30 days), Celery dosage (< 500 mg/day, 500–1,000 mg/day, >1,000 mg/day), Celery part used (celery seeds vs. other parts). Meta-regression was conducted to assess the potential impact of plant part used, dosage, treatment duration, and gender distribution [male-dominant (≥60% male), female-dominant ( ≤ 40% male), and gender-balanced (40% < male proportion < 60%)] on outcomes and to identify sources of heterogeneity. Sensitivity analysis was performed to determine the influence of individual studies on the overall effect size. Publication bias was assessed using funnel plots, Egger's test, and the Begg-Mazumdar correlation test. If publication bias was detected, the “trim-and-fill” method was used for correction. A *p*-value < 0.05 was considered statistically significant in all analyses.

## 3 Results

### 3.1 Study selection

**Figure 1:**
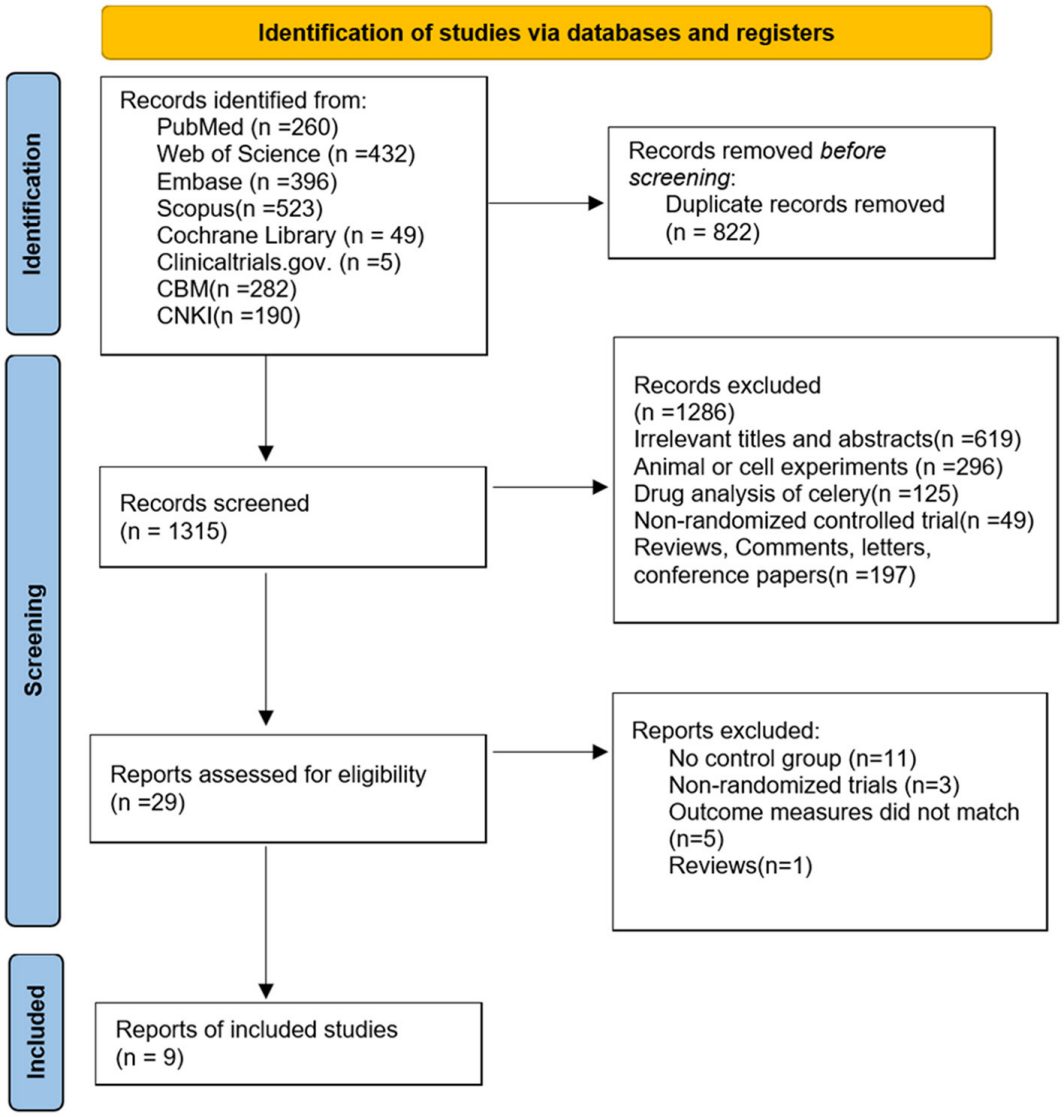
PRISMA flow diagram of selecting studies.

The process of literature screening was based on PRISMA guidelines (18) and is shown in Figure 1. We searched six databases, identifying 2,137 studies. After removing 822 duplicates, we excluded 1,286 studies based on title and abstract screening for the following reasons: irrelevant titles and abstracts (*n* = 619), animal or cell-based studies (*n* = 296), pharmacological analyses of celery (*n* = 125), non-randomized controlled trials (*n* = 49), and reviews, commentaries, letters (*n* = 197). The full texts of the remaining 29 articles were assessed for eligibility. Among the 29 articles, 3 studies were not RCTs (27–29), 11 studies lacked a control group (30–40), and 5 studies did not meet the predefined outcome criteria (41–45), which are therefore excluded. Additionally, one review article was excluded (46). Ultimately, nine studies comprising ten intervention groups were included (one study investigated two different doses of celery, analyzed as separate groups).

### 3.2 Study characteristics and drug safety

This meta-analysis is based on a total of nine randomized controlled trials published between 2002 and 2024, with studies conducted in Iran (15, 17, 47–50), Indonesia (14, 16), and China (51), with the majority originating from Iran. These trials involved participants with hypertension, polycystic ovary syndrome, prediabetes, or overweight conditions. The total sample size was 511 participants. Of the included studies, some were crossover trials (15, 17, 48), while the remaining studies employed parallel control design. Regarding study design, three trials used pharmacological control groups (47, 50, 51), while others used placebo or blank controls. One study had a mean participant age of 26.5 years (47), whereas in the remaining studies, the mean participant age ranged from 50 to 70 years. Among all studies, one trial exclusively recruited female participants (47), one study did not disclose gender distribution, and the remaining studies included both genders.

Celery was administered either as capsules or as decoctions, with capsule doses ranging from 10.55 mg to 2,250 mg per day, and treatment durations varying from 12 to 84 days. Among the ten included intervention groups, eight assessed SBP, eight evaluated DBP, six measured FPG and TC, four examined LDL-c, and three analyzed HDL-c and TG. Further inquiries were made to the corresponding authors via email regarding missing data, but no responses were received. The detailed characteristics of the included trials are presented in Table 1.

**Table 1:** Characteristics of included randomized controlled trials investigating celery interventions.

**References**	**Country/ population**	**Number of** **Participants** **(T/C)**	**Study design**	**Control group type**	**Mean age**	**Gender** **(M/F)**	**Intervention type and Dosage(mg)**	**Duration** **(day)**	**Outcomes**
Supari (14)	Indonesia/hypertension	142 (72/70)	parallel	Amlodipine	60.315	M/F (106/36)	Celery extract capsule, 562.5 mg daily	84 days	SBP, DBP, TG, FPG, TC, HDL-c, LDL-c
Li et al. (51)	China/hypertension	40(20/20)	parallel	Blank control	62.04	M/F (23/17)	Celery stem and leaf decoction, 250g celery cooked (about 187.5 mg celery powder daily) ^*^	30 days	SBP, DBP
Jazani et al. (47)	Iran/PCOS	72(36/36)	parallel	Metformin	26.5	F (72)	Celery seed powder capsule, 2,250 mg daily	15 days	FPG
Yusni et al. (16)	Indonesia/pre-diabetes	16(8/8)	parallel	placebo	68	M/F (6/10)	Celery leaf powder capsules, 750 mg daily	12 days	FPG
Shayani Rad et al. (17)	Iran/ hypertension	51(25/26)	cross- over	placebo	51.275	M/F (25/26)	Celery seed extract capsule, 1,340 mg daily	28 days	SBP, DBP, TG, FPG, TC, HDL-c, LDL-c
Shayani Rad et al. (48)	Iran/ hypertension	52(26/26)	cross- over	placebo	50.515	M/F (26/26)	Celery seed extract capsule, 1,340 mg daily	28 days	SBP, DBP, TG, FPG, TC, HDL-c, LDL-c
Mohsenpour et al. (49)	Iran/over- weight	36(18/18)	parallel	placebo	56.25	M/F (13/23)	Celery stem and leaf powder capsule, 750 mg daily	84 days	SBP, DBP, TG, FPG, TC, HDL-c, LDL-c
Rad et al. (15)	Iran/ hypertension	50(25/25)	cross- over	placebo	50.34	M/F (24/26)	Celery seed extract capsule,1340 mg daily	28 days	SBP, DBP
Febriza et al. (50)	Indonesia/ hypertension	74(46/28)	parallel	Antihypertensive drugs	50.62	M/F (18/56)	Celery stem and leaf decoction, 100 g celery cooked into 250 ml/150 ml decoction (about 17.58 mg/10.55 mg celery powder daily)^*^	30 days	SBP, DBP, TC

T, Treatment group; C, control group; M, male; F, female; NR, not reported; mL, milliliter; SBP, systolic blood pressure; DBP, diastolic blood pressure; TG, triglyceride; FPG, fasting plasma glucose; TC, total cholesterol; HDL-c, high density lipoprotein cholesterol; LDL-c, low density lipoprotein cholesterol; PCOS, polycystic ovary syndrome. For studies where the intervention was a decoction, in addition to reporting the original dose of celery used in the study, the amount of celery extract converted to a constant weight minus water was also reported. The conversion method was derived from the extraction procedure and preparation details of the celery capsule described in the study by Yusni et al. (16).

Regarding safety outcomes, six trials reported adverse events or safety outcomes associated with celery or placebo, while three studies did not provide safety information (16, 50, 51). Of these six trials, three reported only adverse events, whereas the other three reported both adverse events and safety-related laboratory parameters. No significant differences were observed between celery and placebo in terms of severe adverse events, and there was no notable difference in mild adverse events between celery and control groups. Given the wide variety but low frequency of reported adverse events, a descriptive analysis was performed. Comprehensive safety data details are provided in Table 2.

**Table 2:** Adverse reactions and safety indicators.

**References**	**Adverse events**	**Safety indicators**
Supari (14)	No statistical difference between the two groups in terms of all side effects. The most common side effect was dizziness T/C (14/14). Other side effects included weakness T/C (3/1), decreased libido T/C (1/1), flushing T/C (1/0), nausea T/C (1/4), drowsiness T/C (1/2), and increased heart rate T/C (2/2).?	NR
Li et al. (51)	NR	NR
Jazani et al. (47)	No serious adverse reactions. No statistical difference between the two groups in terms of mild adverse reactions. Types of side effects: constipation T/C (1/0), stomach discomfort T/C (1/0), punctate bleeding T/C (1/0), abdominal pain T/C (0/1), nausea T/C (0/1), vaginal bleeding T/C (0/1).	NR
Yusni et al. (16)	NR	NR
Shayani Rad et al. (17)	No significant difference in the number of serious and mild adverse reactions between the two groups (*p >* 0.05). Types of side effects: gastric reflux T/C (2/1), skin irritation T/C (1/0), swelling T/C (1/0), nausea T/C (1/1).	Compared with placebo, celery improved BUN and SCr (*p <* 0.05). No significant differences in liver function, SGOT, SGPT, and ALP between the two groups (*p >* 0.05).
Shayani Rad et al. (48)	No significant difference in the serious and mild adverse reactions between the two groups(*p >* 0.05).Types of side effects: gastric reflux T/C (2/1), headache T/C (1/2), flushing T/C (1/2), dizziness T/C (0/1), skin irritation T/C (1/0), swelling T/C (1/0), nausea T/C (1/1), abdominal pain T/C (1/1), constipation T/C (0/1), fatigue T/C (0/1), and increased heart rate T/C (1/1).	Compared with placebo, celery improved renal function, BUN, ALT, AST, SGPT and SGOT (*p <* 0.05). No significant difference in ALP values between the two groups (*p >* 0.05).
Mohsenpour et al. (49)	Participants reported no side effects.	No significant difference in ALT and AST between the celery group and the placebo group.
Rad et al. (15)	No significant difference in the serious and mild adverse reactions between the two groups (*p >* 0.05). Mild adverse reactions included headache T/C(1/2), skin irritation T/C(1/0), swelling T/C(1/0), abdominal pain T/C(1/1), and constipation T/C(0/1).	NR
Febriza et al. (50) (250/150ml)	NR	NR

T, Treatment group; C, control group; NR, not reported; mL, milliliter; BUN, blood urea nitrogen; SCr, serum creatinine; SGOT, serum glutam-ic-oxaloacetic transaminase; SGPT, Serum Glutamic-Pyruvic Transaminase; ALP, Alkaline Phosphatase; ALT, alanine aminotransferase; AST, aspartate aminotransferase.

### 3.3 Risk of bias assessment

Among all included RCTs, only one study (47) exhibited a low risk of bias across all domains and was considered high-quality. In another study (14), the dropout rate exceeded 10%, with participants withdrawing due to perceived unsuitability of the intervention, leading to its classification as high-risk. The remaining studies are classified as having “some concerns.”

**Figure 2:**
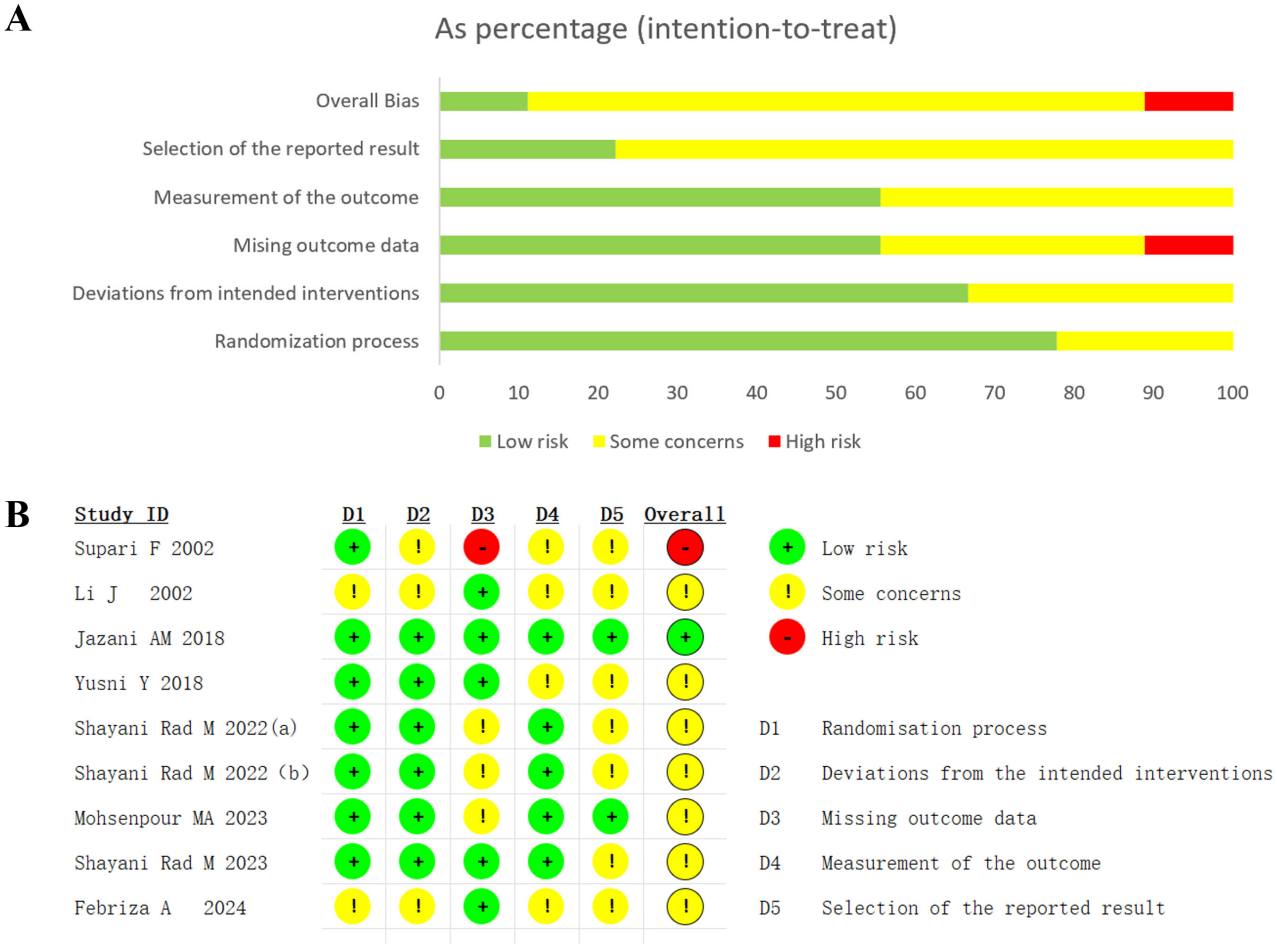
Summary plot of Cochrane Collaboration's risk of bias tool 2 of **(A)** Risk of bias summary. **(B)** Risk of bias diagram.

Among the nine studies, two were open-label trials (50, 51) while the others employed a double-blind design. Three studies explicitly employed block randomization (16, 47, 49), and an additional three utilized random number tables for allocation (15, 17, 48). The remaining studies did not provide detailed descriptions of their randomization methods, potentially impacting the risk of selection bias. Regarding allocation concealment, five studies adopted opaque packaging or sealed envelopes (15, 17, 47–49), while the others did not report allocation concealment procedures, thus limiting the assessment of potential bias in this domain. Details of the randomization and blinding methods are shown in Supplementary Table S2. The full results of the quality assessment are presented in Figure 2.

GRADE assessments (Supplementary Table S3.2) indicated that three outcomes were supported by high-quality evidence, including the non-pharmacological control subgroup for TG and HDL-C, and the pharmacological control subgroup for FPG. Moderate-quality evidence was identified for eight outcomes, including the non-pharmacological control subgroups for SBP, DBP, FPG, and TC, as well as the pharmacological control subgroups for TC, DBP, and HDL-C. Three outcomes were rated as low-quality evidence: the non-pharmacological control subgroup for LDL-C, and the pharmacological control subgroups for SBP and LDL-C. A total of 42.86% of outcomes were downgraded due to inconsistency, primarily driven by the high risk of bias and substantial heterogeneity in the study by Supari (14).

### 3.4 Meta-analysis

**Figure 3:**
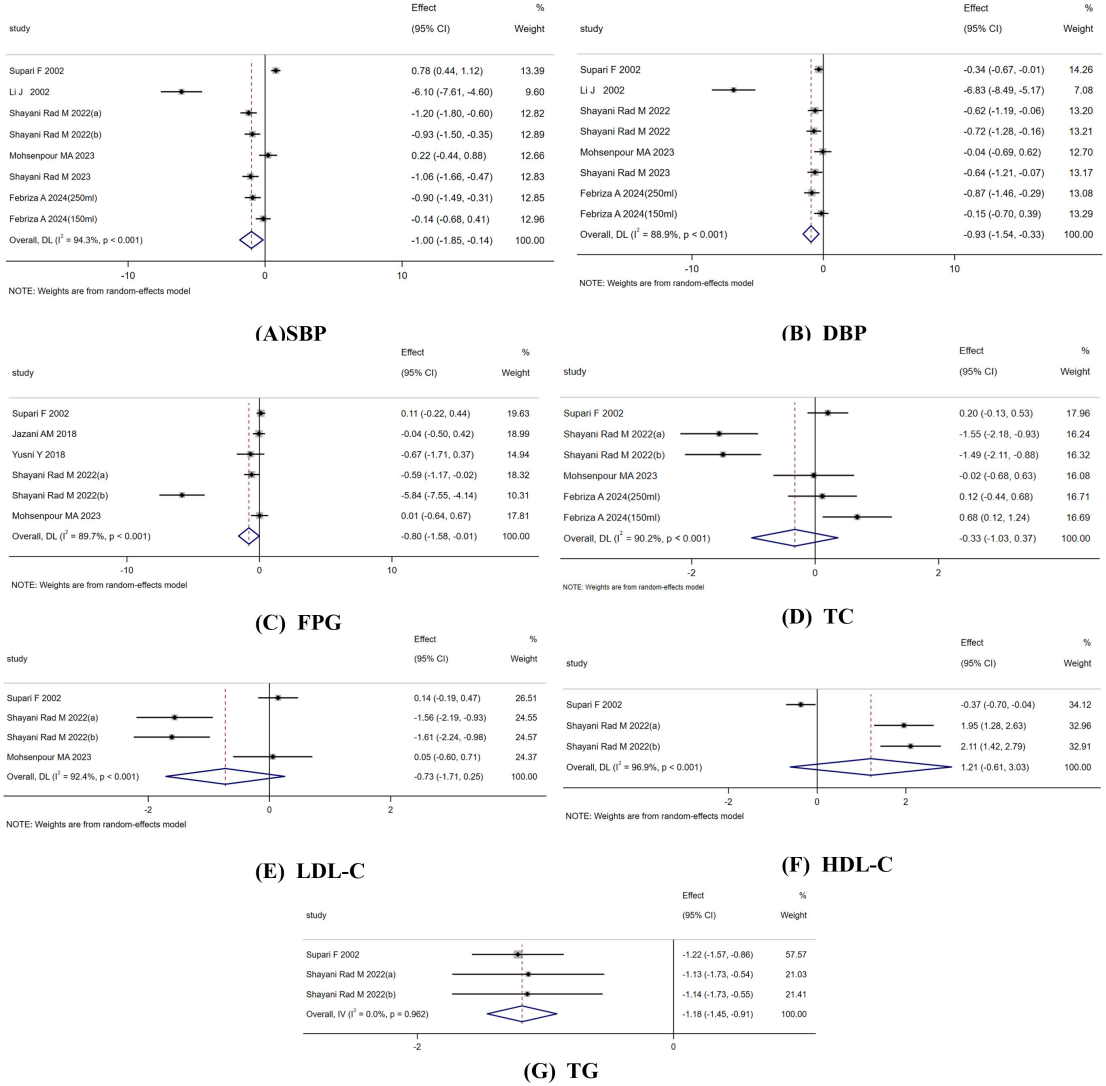
Forest plot reporting WMD and 95%CI for the effects of celery intake on **(A)** systolic blood pressure (SBP). **(B)** Diastolic blood pressure (DBP). **(C)** Fasting plasma glucose (FPG). **(D)** total cholesterol (TC). **(E)** Low-density lipoprotein cholesterol (LDL-c). **(F)** High-density lipoprotein cholesterol (HDL-c). **(G)** Triglycerides (TG).

Eight studies (total sample size = 473) evaluated the efficacy of celery on SBP. The random-effects model revealed that, celery significantly reduced SBP levels compared to the control group (SMD: −1.0; 95% CI: −1.85 to −0.14; *p* = 0.022), with substantial heterogeneity (I^2^ = 94.3%, *p* < 0.001) (Figure 3A). Subgroup analysis indicated the medicinal part of celery, intervention dosage and intervention duration were the sources of heterogeneity (I^2^ has decreased to 0.0%). Meanwhile, subgroup findings indicated celery seeds were more effective than other parts of the plant. Additionally, shorter intervention durations (< 30 days) demonstrated greater efficacy (Table 3).

**Table 3:** Subgroup analyses of celery intake on parameters.

**Parameter**	**Subgroup**	**Number of studies**	**WMD (95%CI)**	**Weight (%)**	***p* within group**	**I^2^ (%)**	***p* heterogeneity**
**SBP**							
	Overall	8	−1.00(−1.85, −0.14)	100	0.022	94.3%	< 0.001
**Control group**							
	Drug	3	−0.06(−1.07, 0.94)	39.20	0.901	92.3%	< 0.001
	Others^*^	5	−1.61(−2.78, −0.45)	60.80	0.007	93.1%	< 0.001
**Intervention type**							
	Capsule	5	−0.43(−1.33, 0.48)	64.59	0.354	93.1%	< 0.001
	Decoction	3	−2.22(−4.43, −0.01)	35.41	0.049	96.3%	< 0.001
**Plant parts used**							
	Seeds	3	−1.06(−1.40, −0.72)	38.54	< 0.001	0.0	0.812
	Others	5	−1.02 (−2.28, 0.25)	61.46	0.116	95.7	< 0.001
**Dosage(mg/day)**							
	< 200	3	−2.22(−4.43, −0.01)	35.41	0.049	96.3	< 0.001
	200–1000	2	0.57(0.04, 1.10)	26.05	0.034	54.7	0.137
	>1000	3	−1.06(−1.40, −0.72)	38.54	< 0.001	0.0	0.812
**Duration (day)**							
	< 30	3	−1.06 (−1.40, −0.72)	38.54	< 0.001	0.0	0.812
	≥30	5	−1.02 (−2.28, 0.25)	61.46	0.116	95.7	< 0.001
**DBP**							
	Overall	8	−0.93 (−1.54, −0.33)	100	0.003	100%	< 0.001
**Control group**							
	Drug	3	−0.42 (−0.78, −0.07)	40.63	0.020	41.6%	0.18
	Others^*^	5	−1.47 (−2.60, −0.33)	59.37	0.011	92.9%	< 0.001
**Intervention**							
	Capsule	5	−0.45(−0.67, −0.23)	66.55	< 0.001	0.0%	0.460
	Decoction	3	−2.42 (−4.73, −0.11)	33.45	0.040	96.5%	< 0.001
**Plant parts used**							
	Seeds	3	−0.66 (−0.99, −0.34)	39.59	< 0.001	0.0	0.967
	Others	5	−1.29 (−2.33, −0.24)	60.41	0.016	93.6	< 0.001
**Dosage(mg/day)**							
	< 200	3	−2.42(−4.73, −0.11)	33.45	0.040	96.5	< 0.001
	200–1,000	2	−0.28(−0.57, 0.02)	26.96	0.064	0.0	0.417
	>1,000	3	−0.66(−0.99, −0.34)	39.59	< 0.001	0.0	0.967
**Duration(day)**							
	< 30	3	−0.66 (−0.99,−0.34)	39.59	< 0.001	0.0	0.967
	≥30	5	−1.29 (−2.33,−0.24)	60.41	0.016	93.6	< 0.001
**Subgroup analysis for FPG**							
	Overall	6	−0.80(−1.58, −0.01)	100	0.046	89.7%	< 0.001
**Control group**							
	Drug	2	0.06 (−0.21,0.33)	38.61	0.658	0.0	0.597
	Others^*^	4	−1.55(−3.10, −0.00)	61.39	0.05	92.4%	< 0.001
**Intervention type**							
	Capsule	6	−0.80(−1.58, −0.01)	100	0.046	89.7%	< 0.001
**Plant parts used**							
	Seeds	3	−1.88(−3.74, −0.01)	47.62	0.049	95.2	< 0.001
	Others	3	0.04(−0.25, 0.32)	52.38	0.804	0.0	0.372
**Dosage(mg/day)**							
	200–1000	3	0.04(−0.25,0.32)	52.38	0.804	0.0	0.372
	>1000	3	−1.88(−3.74, −0.01)	47.62	0.049	95.2	< 0.001
**Duration(day)**							
	< 30	4	−1.52 (−2.95, −0.10)	62.57	0.036	92.8	< 0.001
	≥30	2	0.09(−0.20,0.39)	37.43	0.539	0.0	0.792
**Subgroup analysis for TC**							
	Overall	6	−0.33 (−1.03,0.37)	100	0.355	90.2%	< 0.001
**Control group**							
	Drug	3	0.30 (0.00, 0.59)	51.36	0.049	19.7%	0.288
	Others^*^	3	−1.03 (−1.99,−0.06)	48.64	0.037	85.7%	< 0.001
**Intervention type**							
	capsule	4	−0.70(−1.67, 0.27)	66.60	0.157	92.3%	< 0.001
	decoction	2	0.40 (−0.16,0.95)	33.40	0.158	48.8%	0.162
**Plant parts used**							
	Seeds	2	−1.52 (−1.96,−1.08)	32.57	< 0.001	0.0	0.891
	Others	4	0.25 (−0.00,0.49)	67.43	0.054	7.1	0.358
**Dosage (mg/day)**							
	< 200	2	0.40 (−0.16,0.95)	33.40	0.158	48.8	0.162
	200–1,000	2	0.16(−0.14,0.45)	34.04	0.291	0.0	0.542
	>1,000	2	−1.52(−1.96, −1.08)	32.57	< 0.001	0.0	0.891
**Duration(day)**							
	< 30	2	−1.52 (−1.96,−1.08)	32.57	< 0.001	0.0	0.891
	≥30	4	0.25 (−0.0,0.49)	67.43	0.054	7.1	0.358

^*^Others include placebo and blank control.

Eight studies (total sample size = 473) assessed the impact of celery on DBP. The random-effects model showed that celery significantly reduced DBP levels compared to the control group (SMD: −0.93; 95% CI: −1.54 to −0.33; *p* = 0.003), with considerable heterogeneity (I^2^ = 88.9%, *p* < 0.001) (Figure 3B). Subgroup analysis found the medicinal part of celery, intervention type, intervention dosage and intervention duration were the sources of heterogeneity (I^2^ has decreased to 0.0%). Meanwhile, except for doses between 200 mg and 1,000 mg, which did not show a significant therapeutic effect, whereas all other subgroups exhibited a clear reduction effect (Table 3).

Six studies (total sample size = 369) analyzed the effect of celery on FPG. The random-effects model demonstrated a significant reduction in FPG levels compared to the control group (SMD: −0.80; 95% CI: −1.58 to −0.01; *p* = 0.046), with notable heterogeneity (I^2^ = 89.7%, *p* < 0.001) (Figure 3C). Subgroup analysis showed that heterogeneity decreased when stratified by control type, medicinal part of celery, intervention dosage, and intervention duration (I^2^ has decreased to 0.0%). Celery was more effective in lowering FPG when celery seeds were used, treatment duration was < 30 days, and dosage exceeded 1,000 mg/day.

Six studies (total sample size = 383) evaluated the effect of celery on TC. The random-effects model revealed no significant difference between celery and the control group (SMD: −0.33; 95% CI: −1.03 to 0.37; *p* = 0.355), with substantial heterogeneity (I^2^ = 77.4%, *p* < 0.001) (Figure 3D). Subgroup analysis showed a reduction in heterogeneity when studies were grouped based on medicinal part of celery, intervention dosage and intervention duration (I^2^ has decreased to 0.0%). Celery was more effective when celery seeds were used, the intervention lasted < 30 days, and the dosage exceeded 1,000 mg/day (Table 3).

Four studies (total sample size = 281) reported the effects of celery on LDL-C. Results from the random-effects model indicated no significant difference between celery and the control group (SMD: −0.73; 95% CI: −1.71 to 0.25; *p* = 0.146), with significant heterogeneity (I^2^ = 92.4%, *p* < 0.001) (Figure 3E).

Three studies (total sample size = 245) evaluated the impact of celery on HDL-C. The random-effects model showed no significant difference between celery intervention and placebo (SMD: 1.21; 95% CI: −0.61 to 3.03; *p* = 0.191), with considerable heterogeneity (I^2^ = 96.9%, *p* < 0.001) (Figure 3F).

Three RCTs (total sample size = 245) assessed the effect of celery on TG. The fixed-effects model indicated that celery significantly reduced TG levels compared to the control group (SMD: −1.18; 95% CI: −1.45 to−0.91; *p* < 0.001), with low heterogeneity (I^2^ = 0.0%, *p* = 0.962) (Figure 3G).

### 3.5 Sensitivity analysis

**Figure 4:**
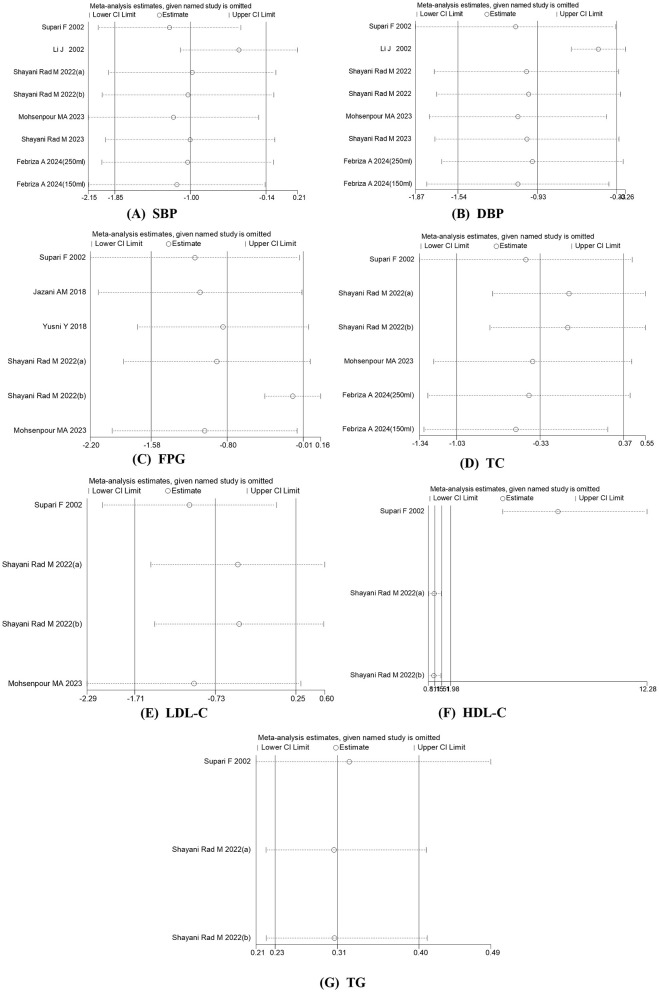
Sensitivity analysis was performed by removing each study in turn to determine the impact of each study on the overall effect size.

To determine the impact of each study on the overall effect size, a leave-one-out sensitivity analysis was conducted. Each study was excluded individually, and the overall effect sizes were recalculated. Our analysis revealed that no single study had a significant influence on the overall effect sizes of SBP, DBP, FPG, TC, LDL-C, or TG. In contrast, the sensitivity analysis for HDL-C indicated that the overall effect size was substantially influenced by the study conducted by Supari (14) (SMD: 7.61; 95% CI: 4.71 to 12.28) (Figure 4).

### 3.6 Meta-regression

**Figure 5:**
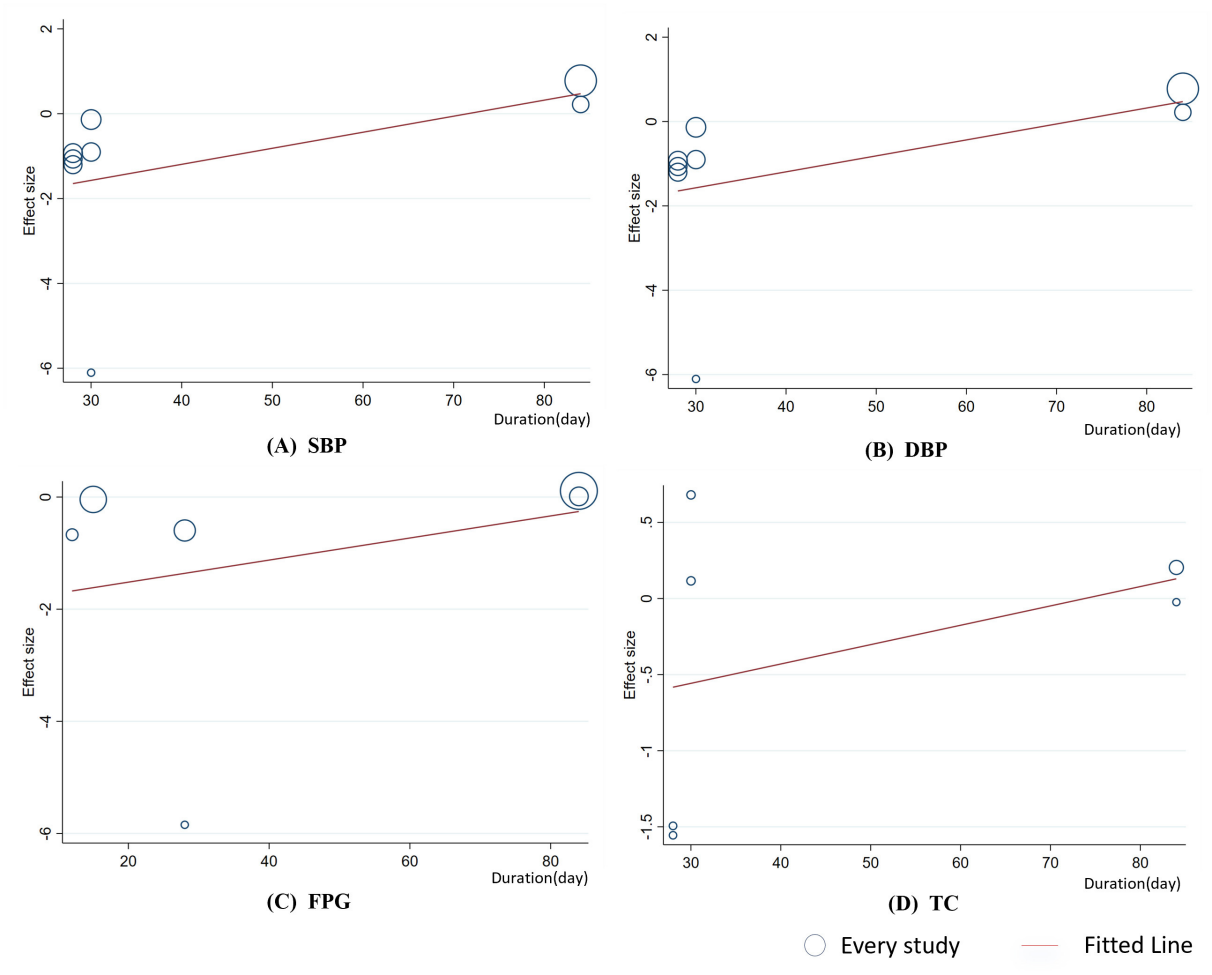
Meta-regression analysis between celery duration and mean difference in **(A)** SBP; **(B)**DBP; **(C)** FPG; **(D)** TC.

**Figure 6:**
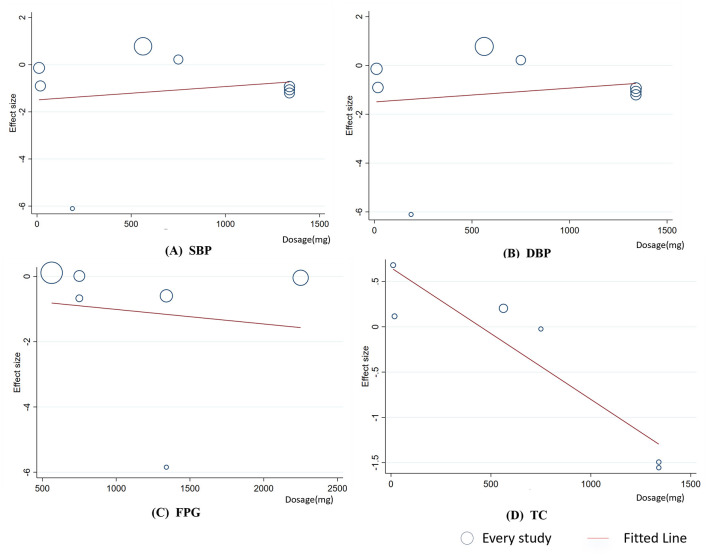
Meta-regression analysis between celery dosage and mean difference in **(A)** SBP; **(B)**DBP; **(C)** FPG; **(D)** TC.

**Figure 7:**
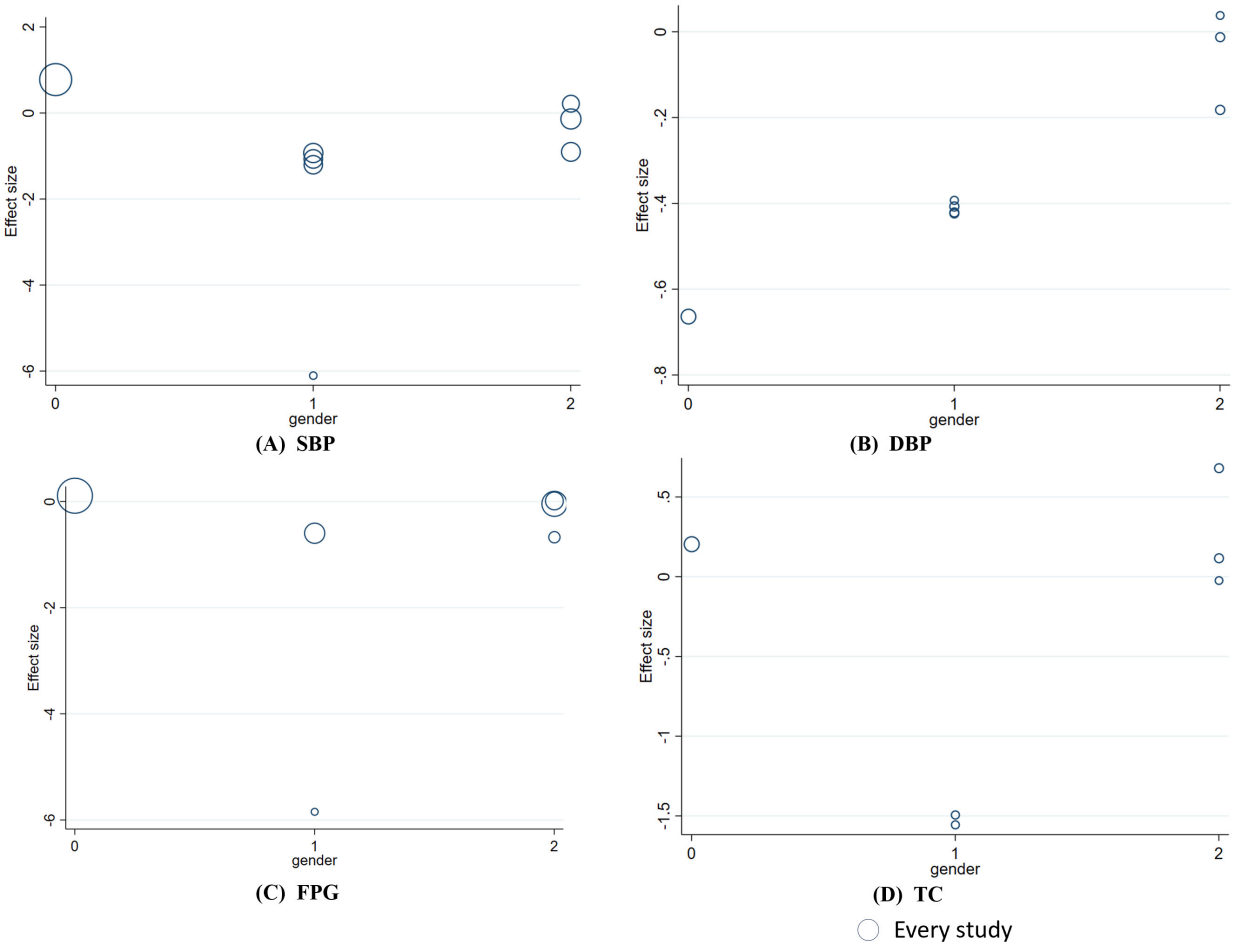
Meta-regression analysis between gender and mean difference in **(A)** SBP; **(B)** DBP; **(C)** FPG; **(D)** TC. 0 indicates male-dominant studies, 1 indicates gender-balanced studies, and 2 indicates female-dominant studies.

**Figure 8:**
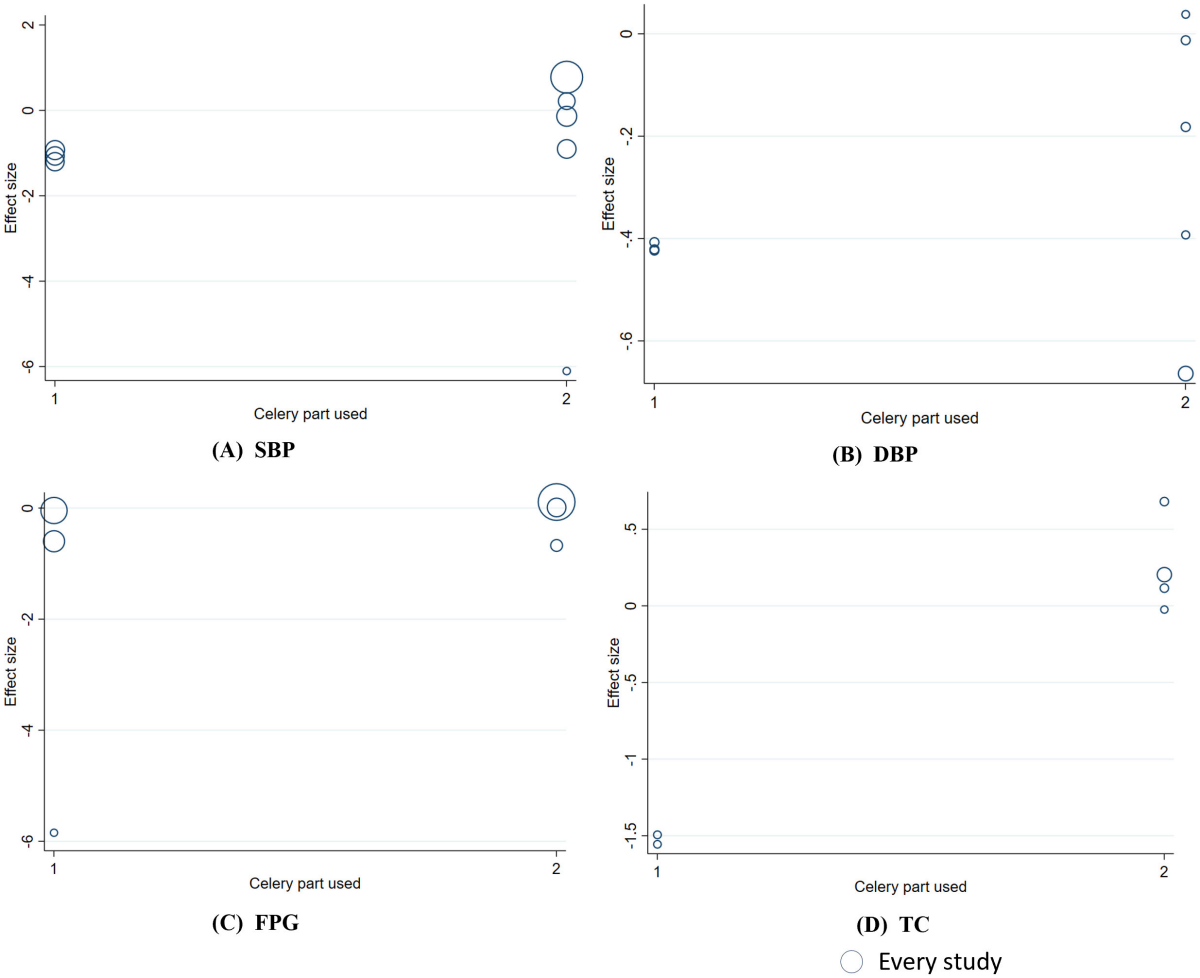
Meta-regression analysis between celery part used and mean difference in **(A)** SBP; **(B)** DBP; **(C)** FPG; **(D)** TC.1 indicates celery seeds, and 2 indicates other parts of the celery plant.

To ensure the robustness of the findings and identify potential sources of heterogeneity, linear regression analysis was performed on outcomes with at least six studies to assess the effects of celery dosage and intervention duration. The analysis revealed no significant linear relationship between intervention duration and changes in SBP, DBP, FPG, or TC (*p*_linear_ > 0.05, Figure 5). However, meta-regression indicated a significant negative linear relationship between intervention dosage and TC levels (Coef. = −0.00145, *p*_linear_ = 0.012). This relationship was not significant for SBP, DBP, or FPG (Figure 6). In addition, DBP was significantly associated with gender (Coef. = 0.26, *p* = 0.047). The heterogeneity in TC was significantly related to the medicinal part of celery used (Coef. = −1.77, *p* = 0.002). No significant associations were observed between the plant part or gender stratification and other outcomes (Figures 7, 8).

### 3.7 Publication bias

Potential publication bias was assessed using funnel plots, Egger's test, and Begg's test. Visual inspection of the funnel plots (Supplementary Figure S4.1) indicated some asymmetry. However, Egger's test and Begg's test (Supplementary Figures S4.2, S4.3) did not detect significant bias for DBP, FPG, TC, or LDL-C, suggesting stable effect estimates for these outcomes. For SBP, HDL-C, and TG, Begg's test did not indicate significant publication bias, but Egger's test suggested potential bias. Therefore, The Trim and Fill method was used for correction. The effect size after SBP correction was (SMD: −0.367; 95% CI: −0.867 to −0.157; *p* = 0.022), the effect size after HDL-C correction was (SMD: −0.691; 95% CI: −0.867 to 0.157; *p* = 0.686), and the effect size after TG correction was (SMD: −0.297; 95% CI: −0.307 to −0.236; *p* < 0.01).

## 4 Discussion

Systematic reviews and meta-analyses help assess the efficacy and safety of dietary supplements, providing valuable insights into their routine incorporation into the daily diets for cardiometabolic health management. Despite traditional use of celery in many cultures for health benefits (6–8), comprehensive evidence synthesis has been lacking. This systematic review and meta-analysis consolidate the latest evidence on the effects of celery on blood pressure, blood sugar, and blood lipid parameters, analyzing nine trials comprising ten studies with 511 participants. Our findings indicate that celery significantly reduces SBP (SMD: −1.0; 95% CI: −1.85 to −0.14; *p* = 0.022), DBP (SMD: −0.93; 95% CI: −1.54 to −0.33; *p* = 0.003), FPG (SMD: −0.80; 95% CI: −1.58 to −0.01; *p* = 0.046), and TG levels (SMD: −1.18; 95% CI: −1.45 to −0.91; *p* < 0.001), while effects on TC, LDL-C, and HDL-C were not statistically significant. Regarding safety, no significant difference was observed between celery and placebo in terms of adverse events.

Most studies and reviews support the blood potential pressure-lowering effects of celery (8, 15, 17, 46, 48). A recent clinical trial (50) found that adequate doses of celery can reduce blood pressure, which aligns with our meta-analysis. In contrast, Mohsenpour et al. (49) reported no significant difference between celery and placebo, underscoring the need for more studies to determine the true effect. Despite the challenge posed by these divergent findings, the significant pooled effect of celery on blood pressure remains noteworthy because even modest reductions in blood pressure may have substantial clinical significance. Epidemiological data from Japanese populations indicate that a sustained 2 mmHg reduction in average SBP can lower the incidence of stroke and ischemic heart disease by approximately 6% and 5%, respectively (52). A study by Dena Ettehad et al. further confirmed that even small decreases in blood pressure are associated with proportionate reductions in cardiovascular events, coronary heart disease, stroke, and heart failure (53). Therefore, in specific clinical settings, celery interventions may offer practical value. Additionally, as a low-cost and highly accessible complementary strategy, celery has the potential to help bridge treatment gaps in resource-limited regions.

Evidence regarding celery's hypoglycemic effect remains inconclusive. While some studies (17, 48) have reported significant glucose-lowering effects, others (47, 49) did not observe significant improvements. From a pharmacological perspective, celery seeds are rich in 3-butylphthalide, which can lower blood sugar levels and improve insulin tolerance (10). Although the pooled effect size for blood glucose in our study indicated a beneficial effect, the effect size was near the line of null effect, suggesting a marginally significant impact. Therefore, further large-scale, high-quality studies are needed to clarify the clinical relevance of celery's hypoglycemic action.

Clinical trials examining the effects of celery on lipid profiles are limited and show inconsistent results. Recent studies (47, 49) reported no significant impact on TC. However, studies by Shayani Rad et al. (17, 48) demonstrated that celery seeds significantly improved several lipid parameters, including TC, HDL-C, LDL-C, and TG. Animal and mechanistic studies provide additional evidence supporting celery's lipid-lowering potential. For instance, Ahmed (54) reported that celery seed improved lipid profiles, potentially through mechanisms such as inhibiting hepatic cholesterol synthesis, enhancing lecithin–cholesterol acyltransferase activity, and reducing intestinal lipid absorption. Zhao et al. (55) showed that fermented celery juice alleviated dyslipidemia and visceral fat accumulation in mice fed a high-fat diet. Other animal studies (56, 57) have shown that celery decreases serum LDL, TC, and TG levels. In our meta-analysis, celery significantly reduced triglyceride (TG) levels. However, no significant improvements were observed for TC, HDL-C, or LDL-C. Future research should focus on populations with specific dyslipidemias to better evaluate celery's effects on lipid profiles.

In addition, publication bias was detected in the significant outcomes of DBP and TG; however, the results remained statistically significant after adjustment using the trim-and-fill method. Nevertheless, these outcomes should still be interpreted with caution given the presence of publication bias.

Our subgroup and meta-regression analyses revealed several important patterns regarding intervention characteristics and efficacy:

**1. Control intervention type**: the therapeutic effects of celery varied depending on the type of control intervention. When compared with non-pharmacological interventions (e.g., Placebo and blank control), celery demonstrated significant therapeutic effects on blood pressure, FPG, and TC, highlighting its potential as a dietary intervention for prevention. For blood pressure outcomes, celery showed no statistically significant difference compared to pharmacologic treatments, and even demonstrated a greater reduction in DBP. Studies have shown that celery can induce vasodilation in aortic endothelial cells, potentially lowering blood pressure through mechanisms such as inhibition of receptor-operated and voltage-dependent calcium channels, release of endothelium-derived hyperpolarizing factors, and activation of voltage-dependent potassium channels (58).
**2. Plant part used**: subgroup analysis indicated that the therapeutic effects of celery differed by the plant part used. Interventions using celery seeds showed significant improvements in blood pressure, FPG, and TC. In contrast, preparations using other parts of the plant (e.g., stalks and leaves) only showed a significant reduction in DBP. Previous research demonstrates compositional differences among celery parts (6, 59–61). Celery seeds are rich in flavonoids, phthalides (e.g., sedanolide, 3-n-butylphthalide), and monoterpenes (e.g., limonene), whereas celery stalks and leaves contain higher levels of phenolic acids [e.g., chlorogenic acid and ferulic acid (62)], furanocoumarins (e.g., 5-hydroxy and 8-hydroxy methoxyfuranocoumarins), and flavonoids (e.g., apigenin, quercetin). These compounds may mediate distinct pharmacological actions: flavonoids may lower blood pressure and glucose via enzyme inhibition and antioxidant effects (63); phenolic acids are also associated with blood pressure regulation (64); furanocoumarins may exert antihypertensive effects through anti-inflammatory pathways. In celery seeds, the phthalide derivative CD21 has been shown to slow atherosclerosis progression and reduce hypertension by inhibiting AP-1 and NF-κB expression (65). Compared with celery stems and leaves, 3-butylphthalide, which is unique to celery seeds, can lower blood sugar levels and improve insulin tolerance, while inhibiting lipid accumulation and increasing free fatty acid uptake and oxygen consumption rate (10). Limonene activates the AMPK signaling pathway to regulate lipid metabolism (66). Collectively, differences in chemical composition may be one of the potential reasons for the varied effects observed among different parts of the celery plant. Meta-regression for the TC outcome confirmed that plant part was a source of heterogeneity, supporting differential efficacy based on the plant component used.
**3. Dosage effects:** our analysis showed that celery powder doses exceeding 1,000 mg per day were associated with greater efficacy. However, we also observed that doses below 200 mg per day also demonstrated significant therapeutic effects. Notably, these lower-dose interventions primarily involved celery decoctions rather than powdered celery. To ensure dose consistency, we standardized the decoction dose by converting it into an equivalent amount of celery powder. The decoctions were prepared by boiling fresh celery stalks and leaves. Thermal processing of fresh celery can disrupt plant cell walls, thereby enhancing the release and solubility of lipophilic compounds (67). Nevertheless, this standardization may not fully account for differences in phytochemical composition or bioavailability. The studies included in the low- and high-dose subgroups differed in several methodological and design aspects: low-dose studies primarily used other parts of the celery plant, while high-dose studies used celery seeds; low-dose studies were open-label, whereas high-dose studies were blinded. Therefore, the observed efficacy at low doses may be confounded by these differences and should be interpreted with caution.
**4. Intervention duration:** shorter intervention durations (< 30 days) showed better therapeutic effects, which may reflect physiological adaptation or metabolic tolerance with prolonged supplementation (68). This finding suggests that intermittent rather than continuous supplementation might optimize long-term benefits, a hypothesis requiring further investigation.

Some studies (17, 48) reported that celery consumption was associated with mild adverse events such as frequent urination, and gastrointestinal discomfort, although these effects were infrequent in clinical practice. Concerning safety, our study found no significant differences in overall adverse events or safety parameters between celery and placebo. The favorable safety profile suggests celery could be appropriate for long-term use as a dietary supplement.

In the GRADE assessment, the non-pharmacological control group demonstrated moderate to high-quality evidence for blood pressure and glycemic outcomes, with significant effects observed—supporting the potential metabolic benefits of celery intervention. However, evidence downgrades due to high heterogeneity highlight the need to reduce confounding factors. Meanwhile, the RoB 2 assessment identified one study (14) as having a high risk of bias, which directly contributed to the downgrading of GRADE ratings in five pharmacological control group outcomes. This suggests that the vulnerability of evidence in the drug-controlled subgroup is largely attributable to methodological shortcomings. Future research is encouraged to adhere strictly to the CONSORT guidelines, improve methodological reporting, and strengthen quality control.

Despite strict adherence to Cochrane guidelines for literature search and screening, this meta-analysis has some limitations:

(1) The studies included in this meta-analysis generally feature small sample sizes and are all conducted in Asian populations. Although the random-effects model help to some extent in adjusting for this situation, the generalizability of our conclusions may still be limited, particularly concerning potential influencing factors such as genetic background, age and dietary habits. Future research should focus on multi-center, large-scale studies in various geographic regions to validate the robustness of the findings.
(2) Considerable heterogeneity observed in key outcomes. Although our subgroup and meta-regression analyses partially accounted for this variation, some heterogeneity remained unexplained. This may be due to the multifactorial nature of heterogeneity sources and methodological differences across studies. Future research should aim to reduce heterogeneity through methodological standardization (such as celery preparation methods and dosage) and rigorous clinical implementation.
(3) Some studies lacked detailed descriptions of study design, randomization, and blinding, increasing the risk of potential bias.

## 5 Conclusion

Celery significantly improves SBP, DBP, FPG, and TG levels while having no significant effect on TC, HDL-C, or LDL-C, with a favorable safety profile. These findings suggest that celery may have potential value in modulating cardiometabolic parameters. In the prevention and management of clinically relevant conditions, healthcare professionals may consider appropriate supplementation strategies based on individual patient needs. Further research should focus on standardized preparations, defined patient populations, and optimal regimens regarding dosing to maximize long-term benefits.

## Data Availability

The original contributions presented in the study are included in the article/Supplementary material, further inquiries can be directed to the corresponding author.
