# Supplementary material for: Effects of celery (Apium graveolens) on blood pressure, glycemic and lipid profile in adults: a systematic review and meta-analysis of randomized controlled trials
Source: Front Nutr. 2025 Jul 22;12:1597680. doi: 10.3389/fnut.2025.1597680 (PMC12321563; doi:10.3389/fnut.2025.1597680)
Supplement: Supplementary file 1 [file Table_1.docx]

Supplementary Material

# Search strategies for databases

| Database | Search | Search Strings |
| --- | --- | --- |
| PubMed | #1 | ((Apium graveolens)OR (Wild Celery)OR (Celeries, Wild)OR (Celery, Wild)OR (Wild Celeries)OR (Apium graveolens var. dulce)OR (Celery)OR (Celeries)OR (Apium graveolens var. rapaceum)OR (Celeriac)OR (Celeriacs)) AND (((Metabolic Syndromes) OR(Reaven Syndrome X) OR(Syndrome X, Reaven) OR(Insulin Resistance Syndrome X) OR(Metabolic Cardiovascular Syndrome) OR(Cardiovascular Syndrome, Metabolic) OR(Cardiovascular Syndromes, Metabolic) OR(Syndrome X, Insulin Resistance) OR(Syndrome, Metabolic X) OR(Dysmetabolic Syndrome X) OR(Syndrome X, Dysmetabolic) OR(Syndrome X, Metabolic) OR(Cardiometabolic Syndromes) OR(Metabolic Syndrome) OR(MetS) OR(MS) OR(Hyperlipidemia) OR(Hyperlipemia) OR(Hyperlipemias) OR(Lipidemia) OR(Lipidemias) OR(Lipemia) OR(Lipemias) OR(Hyperlipidemias) OR(High Blood Pressure) OR(High Blood Pressures) OR(Hypertension) OR(Hyperglycemias) OR(Hyperglycemia) OR(Obesity) OR(obese) OR(overweight) OR(waist) OR(waist circumference)OR(waist circumferences) OR(Cardiovascular Diseases) OR(Atherosclerosis) OR(atherosclero) OR(arter))) |
| Web of Science | #1 | (TS=((Apium graveolens)OR (Wild Celery)OR (Celeries, Wild)OR (Celery, Wild)OR (Wild Celeries)OR (Apium graveolens var. dulce)OR (Celery)OR (Celeries)OR (Apium graveolens var. rapaceum)OR (Celeriac)OR (Celeriacs))) AND TS=((Metabolic Syndromes) OR(Reaven Syndrome X) OR(Syndrome X, Reaven) OR(Insulin Resistance Syndrome X) OR(Metabolic Cardiovascular Syndrome) OR(Cardiovascular Syndrome, Metabolic) OR(Cardiovascular Syndromes, Metabolic) OR(Syndrome X, Insulin Resistance) OR(Syndrome, Metabolic X) OR(Dysmetabolic Syndrome X) OR(Syndrome X, Dysmetabolic) OR(Syndrome X, Metabolic) OR(Cardiometabolic Syndromes) OR(Metabolic Syndrome) OR(MetS) OR(MS) OR(Hyperlipidemia) OR(Hyperlipemia) OR(Hyperlipemias) OR(Lipidemia) OR(Lipidemias) OR(Lipemia) OR(Lipemias) OR(Hyperlipidemias) OR(High Blood Pressure) OR(High Blood Pressures) OR(Hypertension) OR(Hyperglycemias) OR(Hyperglycemia) OR(Obesity) OR(obese) OR(overweight) OR(waist) OR(waist circumference)OR(waist circumferences) OR(Cardiovascular Diseases) OR(Atherosclerosis) OR(atherosclero) OR(arter)) and Preprint Citation Index (Exclude – Database) |
| EMBASE | #1 | 'apium graveolens'/exp OR 'apium graveolens' OR 'wild celery' OR 'celeries, wild' OR 'celery, wild' OR 'wild celeries' OR 'apium graveolens var. dulce'/exp OR 'apium graveolens var. dulce' OR 'celery'/exp OR 'celery' OR 'celeries' OR 'apium graveolens var. rapaceum'/exp OR 'apium graveolens var. rapaceum' OR 'celeriac'/exp OR 'celeriac' OR 'celeriacs' |
|  | #2 | 'metabolic syndromes' OR 'reaven syndrome x' OR 'syndrome x, reaven' OR 'insulin resistance syndrome x' OR 'metabolic cardiovascular syndrome' OR 'cardiovascular syndrome, metabolic' OR 'cardiovascular syndromes, metabolic' OR 'syndrome x, insulin resistance' OR 'syndrome, metabolic x' OR 'dysmetabolic syndrome x' OR 'syndrome x, dysmetabolic' OR 'syndrome x, metabolic' OR 'cardiometabolic syndromes' OR 'metabolic syndrome' OR 'mets' OR 'ms' OR 'hyperlipidemia' OR 'hyperlipemia' OR 'hyperlipemias' OR 'lipidemia' OR 'lipidemias' OR 'lipemia' OR 'lipemias' OR 'hyperlipidemias' OR 'high blood pressure' OR 'high blood pressures' OR 'hypertension' OR 'hyperglycemias' OR 'hyperglycemia' OR 'obesity' OR 'obese' OR 'overweight' OR 'waist' OR 'waist circumference' OR 'waist circumferences' OR 'cardiovascular diseases' OR 'atherosclerosis' OR 'atherosclero' OR 'arter' |
|  | #3 | #1 AND #2 |
| Scopus | #1 | Search scope：title, abstract, keywords = (Apium graveolens)OR (Wild Celery)OR (Celeries, Wild)OR (Celery, Wild)OR (Wild Celeries)OR (Apium graveolens var. dulce)OR (Celery)OR (Celeries)OR (Apium graveolens var. rapaceum)OR (Celeriac)OR (Celeriacs) |
|  | #2 | Search scope：title, abstract, keywords = (Apium graveolens)OR (Wild Celery)OR (Celeries, Wild)OR (Celery, Wild)OR (Wild Celeries)OR (Apium graveolens var. dulce)OR (Celery)OR (Celeries)OR (Apium graveolens var. rapaceum)OR (Celeriac)OR (Celeriacs))) AND TS=((Metabolic Syndromes) OR(Reaven Syndrome X) OR(Syndrome X, Reaven) OR(Insulin Resistance Syndrome X) OR(Metabolic Cardiovascular Syndrome) OR(Cardiovascular Syndrome, Metabolic) OR(Cardiovascular Syndromes, Metabolic) OR(Syndrome X, Insulin Resistance) OR(Syndrome, Metabolic X) OR(Dysmetabolic Syndrome X) OR(Syndrome X, Dysmetabolic) OR(Syndrome X, Metabolic) OR(Cardiometabolic Syndromes) OR(Metabolic Syndrome) OR(MetS) OR(MS) OR(Hyperlipidemia) OR(Hyperlipemia) OR(Hyperlipemias) OR(Lipidemia) OR(Lipidemias) OR(Lipemia) OR(Lipemias) OR(Hyperlipidemias) OR(High Blood Pressure) OR(High Blood Pressures) OR(Hypertension) OR(Hyperglycemias) OR(Hyperglycemia) OR(Obesity) OR(obese) OR(overweight) OR(waist) OR(waist circumference)OR(waist circumferences) OR(Cardiovascular Diseases) OR(Atherosclerosis) OR(atherosclero) OR(arter) |
|  | #3 | #1 AND #2 |
| Cochrane Library | #1 | (Apium graveolens)OR (Wild Celery)OR (Celeries, Wild)OR (Celery, Wild)OR (Wild Celeries)OR (Apium graveolens var. dulce)OR (Celery)OR (Celeries)OR (Apium graveolens var. rapaceum)OR (Celeriac)OR (Celeriacs) in Title Abstract Keyword |
|  | #2 | (Apium graveolens)OR (Wild Celery)OR (Celeries, Wild)OR (Celery, Wild)OR (Wild Celeries)OR (Apium graveolens var. dulce)OR (Celery)OR (Celeries)OR (Apium graveolens var. rapaceum)OR (Celeriac)OR (Celeriacs))) AND TS=((Metabolic Syndromes) OR(Reaven Syndrome X) OR(Syndrome X, Reaven) OR(Insulin Resistance Syndrome X) OR(Metabolic Cardiovascular Syndrome) OR(Cardiovascular Syndrome, Metabolic) OR(Cardiovascular Syndromes, Metabolic) OR(Syndrome X, Insulin Resistance) OR(Syndrome, Metabolic X) OR(Dysmetabolic Syndrome X) OR(Syndrome X, Dysmetabolic) OR(Syndrome X, Metabolic) OR(Cardiometabolic Syndromes) OR(Metabolic Syndrome) OR(MetS) OR(MS) OR(Hyperlipidemia) OR(Hyperlipemia) OR(Hyperlipemias) OR(Lipidemia) OR(Lipidemias) OR(Lipemia) OR(Lipemias) OR(Hyperlipidemias) OR(High Blood Pressure) OR(High Blood Pressures) OR(Hypertension) OR(Hyperglycemias) OR(Hyperglycemia) OR(Obesity) OR(obese) OR(overweight) OR(waist) OR(waist circumference)OR(waist circumferences) OR(Cardiovascular Diseases) OR(Atherosclerosis) OR(atherosclero) OR(arter) in Title Abstract Keyword |
|  | #3 | #1 AND #2 |
| Clinicaltrials.gov. | #1 | **Condition/disease：**metabolic syndrome or Cardiovascular Syndrome or Hyperlipidemia or Hyperlipemia or High Blood Pressure or Hypertension or Hyperglycemia or Obesity or waist circumferences or Cardiovascular diseases or Atherosclerosis or arter  **Intervention/treatment：**Celery |
| China Biology Medicine disc | #1 | 检索条件:"芹菜“[全部字段:智能\| AND(血脂过多 OR 血脂异常 OR 高脂血症 OR 脂血症 OR 血压过髙 OR 高血压 OR 肥胖症 OR 肥胖OR 体重 OR 超重 OR 多脂症 OR 腰围 OR 高血糖症 OR 血糖过髙 OR 高糖血症 OR 高血糖 OR 高血脂症 OR 代谢综合征 ORX综合征OR 胰岛素抵抗综合征 OR 抗胰岛素性综合征X OR 代谢不良X综合征 OR Reaven X综合征 OR 代谢性心血管综合征 OR 心血管疾病 OF动脉粥样硬化 OR 动脉) |
| China National Knowledge Infrastructure | #1 | (TI%芹菜OR AB%芹菜) AND (TI%血脂过多 OR TI%血脂异常 OR TI%高脂血症 OR TI%脂血症 OR TI%血压过高 OR TI%高血压 OR TI%肥胖症 OR TI%肥胖 OR TI%体重 OR TI%超重 OR TI%多脂症 OR TI%腰围 OR TI%高血糖症 OR TI%血糖过高 OR TI%高糖血症 OR TI%高血糖 OR TI%高血脂症 OR TI%代谢综合征 OR TI%X 综合征 OR TI%胰岛素抵抗综合征 OR TI%抗胰岛素性综合征X OR TI%代谢不良X综合征 OR TI%Reaven X综合征 OR TI%代谢性心血管综合征 OR AB%血脂过多 OR AB%血脂异常 OR AB%高脂血症 OR AB%脂血症 OR AB%血压过高 OR AB%高血压 OR AB%肥胖症 OR AB%肥胖 OR AB%体重 OR AB%超重 OR AB%多脂症 OR AB%腰围 OR AB%高血糖症 OR AB%血糖过高 OR AB%高糖血症 OR AB%高血糖 OR AB%高血脂症 OR AB%代谢综合征 OR AB%X 综合征 OR AB%胰岛素抵抗综合征 OR AB%抗胰岛素性综合征X OR AB%代谢不良X综合征 OR AB%Reaven X综合征 OR AB%代谢性心血管综合征) |

# 2 A detailed description of the randomization and blinding methods included in the study

**Table S2 Randomized and blinded methods included in the study**

| Study | Random Methods | | Blind methods |
| --- | --- | --- | --- |
| Supari F 2002 | Random (not described in detail) | The frequency of oral medication and the size and color of capsules were the same for both groups. | |
| Li J 2002 | Random (not described in detail) | Open label | |
| Jazani AM 2018 | Convenience sampling was used to allocate subjects to the groups, and fixed-size block randomization was used to assign patients to any eligible group. The random sequence was determined by the Randlist 11 software package. | Triple-blind. Drug and placebo were in capsule form with uniform shape, size and color. To conceal allocation, a person outside the research team coded the capsules (A and B), placed them in sealed opaque packages according to the randomization list and numbered them consecutively. This blinded the study (double dummy technique). | |
| Yusni Y 2018 | Simple random sampling method for lottery system | The frequency of oral medication and the size and color of capsules were the same for both groups. | |
| Shayani Rad M 2022(a) | The capsule containers were coded and randomized using a 6-digit number obtained from the "Random Number Table". The first column of the random number table was assigned to the celery washout-placebo group, and the second column was assigned to the placebo washout-celery group. The code was written on a piece of paper and placed in an opaque envelope. The envelopes were sealed and placed in a box in turn, which was kept by the researchers and doctors. | Triple-blind. Celery and placebo capsules were prepared similarly. They were identical in shape, color, size, texture, and smell. The capsules were packaged in identical containers with randomization codes. Therefore, the subjects, researchers, physicians, and data analysts were blinded to the nature of the treatment and placebo groups. | |
| Shayani Rad M 2022(b) |  |  |  |
| Shayani Rad M 2023 |  |  |  |
| Mohsenpour MA 2023 | Block randomization (1:1 ratio) Randomization and concealment sequence were completed by individuals not involved in the study. The allocation envelope was then opened to disclose the assigned group named A or B. | Subjects entered the 12-week study based on their assigned group. The capsules were named A or B within the study group and were similar in appearance, size, and color to conceal the identity of the participants and the researchers. | |
| Febriza A 2024 (250mL/150mL) | Random (not described in detail) | Open label | |

# 3 Details of the GRADE-specific downgrading method and table of results

**3.1 Methods**

The certainty of the evidence is assessed using the GRADE (Grading of Recommendations Assessment, Development, and Evaluation) approach([2012](#_ENREF_1)) (Assessment used online table <https://www.gradepro.org/>). Given the outcome differences between drug-controlled and placebo/blank-controlled groups, the results were presented in separate subgroups. The initial rating for RCTs was considered “high,” and downgrading was performed based on the following domains:

(1) Risk of bias: Assessed using the RoB tool([Saueressig et al., 2022](#_ENREF_7)).

 – No downgrade: if 100% of studies were rated as low risk of bias, or 75–100% were rated as “some concerns”；

 – Downgrade by 1 level: if 50% of studies had high risk of bias；

 – Downgrade by 2 levels: if ≥75% of studies had high risk of bias.

(2) Inconsistency: Based on I² and p-values([Kelu et al., 2020](#_ENREF_6)).

 – No downgrade: if 0% ≤ I² < 50% and p > 0.05;

 – Downgrade by 1 level: if 50% ≤ I² < 99% and p ≤ 0.05;

 – Downgrade by 2 levels: if I² ≥ 99% and p ≤ 0.05.

(3) Indirectness: When the study objectives aligned with the PICO framework, the evidence was considered free from indirectness and was not downgraded. Conversely, if misalignment existed, the evidence was downgraded by one level due to indirectness([Goldkuhle et al., 2023](#_ENREF_4)).

(4) Imprecision: Given that the intervention is a dietary supplement with no serious side effects, convenient use, and low cost([Guyatt et al., 2021](#_ENREF_5)), and even small reductions in relevant indicators may proportionally lower the risk of cardiovascular events, coronary heart disease, stroke, and heart failure([Ettehad et al., 2016](#_ENREF_3)). We defined the minimal clinically important difference as the null value (0). Therefore, if the 95% confidence interval crossed the null line, the certainty was downgraded by one level([2024](#_ENREF_2)).

**3.2 Results**

**Table S3.2 Certainty of Evidence (GRADE approach) of meta-analytic outcomes**

| **Certainty assessment** | | | | | | | **№ of patients** | | | **Effect** | **Certainty** | **Importance** |
| --- | --- | --- | --- | --- | --- | --- | --- | --- | --- | --- | --- | --- |
| **№ of studies** | **Study design** | **Risk of bias** | **Incon-sistency** | **Indirect-ness** | **Impre-cision** | **Other considerations** | **Inter-vention** | **Control** | **Relative (95% CI)** | |  |  |
| **Control group: Non-drug (assessed with: SBP)** | | | | | | | | | | | | |
| 5 | randomised trials | not serious | serious^a^ | not serious | not serious | none | 124 | 115 | | SMD **1.61 SD lower** (2.78 lower to 0.45 lower) | ⨁⨁⨁◯ Moderate^a^ | CRITICAL |
| **Control group: drug (assessed with: SBP)** | | | | | | | | | | | | |
| 3 | randomised trials | serious^b^ | serious^a^ | not serious | not serious | none | 96 | 98 | | SMD **0.06 SD lower** (1.07 lower to 0.94 higher) | ⨁⨁◯◯ Low^a,b^ | IMPORTANT |
| **Control group: Non-drug (assessed with: FPG)** | | | | | | | | | | | | |
| 4 | randomised trials | not serious | serious^a^ | not serious | not serious | none | 77 | 78 | | SMD **1.55 SD lower** (3.1 lower to 0) | ⨁⨁⨁◯ Moderate^a^ | CRITICAL |
| **Control group: drug (assessed with: FPG)** | | | | | | | | | | | | |
| 2 | randomised trials | not serious | not serious | not serious | not serious | none | 108 | 106 | | SMD **0.06 SD higher** (0.21 lower to 0.33 higher) | ⨁⨁⨁⨁ High | NOT IMPORTANT |
| **Control group: Non-drug (assessed with: TC)** | | | | | | | | | | | | |
| 3 | randomised trials | not serious | serious^a^ | not serious | not serious | none | 96 | 98 | | SMD **1.03 SD lower** (1.99 lower to 0.06 lower) | ⨁⨁⨁◯ Moderate^a^ | IMPORTANT |
| **Control group: drug (assessed with: TC)** | | | | | | | | | | | | |
| 3 | randomised trials | serious^b^ | not serious | not serious | not serious | none | 69 | 70 | | SMD **0.3 SD higher** (0 to 0.59 higher) | ⨁⨁⨁◯ Moderate^b^ | IMPORTANT |
| **Control group: Non-drug (assessed with: DBP)** | | | | | | | | | | | | |
| 5 | randomised trials | not serious | serious^a^ | not serious | not serious | none | 124 | 115 | | SMD **1.47 SD lower** (2.6 lower to 0.33 lower) | ⨁⨁⨁◯ Moderate^a^ | CRITICAL |
| **Control group:drug (assessed with: DBP)** | | | | | | | | | | | | |
| 3 | randomised trials | serious^b^ | not serious | not serious | not serious | none | 96 | 98 | | SMD **0.42 SD lower** (0.78 lower to 0.07 lower) | ⨁⨁⨁◯ Moderate^b^ | IMPORTANT |
| **Control group: Non-drug (assessed with: LDL-** | | | | | | | | | | | | |
| 3 | randomised trials | not serious | serious^a^ | not serious | serious^c^ | none | 69 | 70 | | SMD **1.04 SD lower** (2.1 lower to 0.02 higher) | ⨁⨁◯◯ Low^a,c^ | IMPORTANT |
| **Control group: drug (assessed with: LDL-C)** | | | | | | | | | | | | |
| 1 | randomised trials | serious^b^ | not serious | not serious | not serious | none | 72 | 70 | | SMD **0.14 SD higher** (0.19 lower to 0.47 higher) | ⨁⨁⨁◯ Moderate^b^ | NOT IMPORTANT |
| **Control group: Non-drug (assessed with: HDL-C)** | | | | | | | | | | | | |
| 2 | randomised trials | not serious | not serious | not serious | not serious | none | 51 | 52 | | SMD **2.01 SD higher** (1.35 higher to 2.71 higher) | ⨁⨁⨁⨁ High | NOT IMPORTANT |
| **Control group: drug (assessed with: HDL-C)** | | | | | | | | | | | | |
| 1 | randomised trials | serious^b^ | not serious | not serious | not serious | none | 72 | 70 | | SMD **0.37 SD lower** (0.7 lower to 0.04 lower) | ⨁⨁⨁◯ Moderate^b^ | NOT IMPORTANT |
| **Control group: Non-drug (assessed with: TG)** | | | | | | | | | | | | |
| 2 | randomised trials | not serious | not serious | not serious | not serious | none | 51 | 52 | | SMD **1.14 SD lower** (1.56 lower to 0.72 lower) | ⨁⨁⨁⨁ High | IMPORTANT |
| **Control group: drug (assessed with: TG)** | | | | | | | | | | | | |
| 1 | randomised trials | serious^b^ | not serious | not serious | not serious | none | 72 | 70 | | SMD **1.22 SD lower** (1.57 higher to 0.86 lower) | ⨁⨁⨁◯ Moderate^b^ | IMPORTANT |

**CI:** confidence interval; **SMD:** standardised mean difference

Explanations

a. I²≥75% but does not affect the conclusion

b. Supari F 2002 is high risk

c. 95%CI crosses the line

# 4 Supplementary Figures


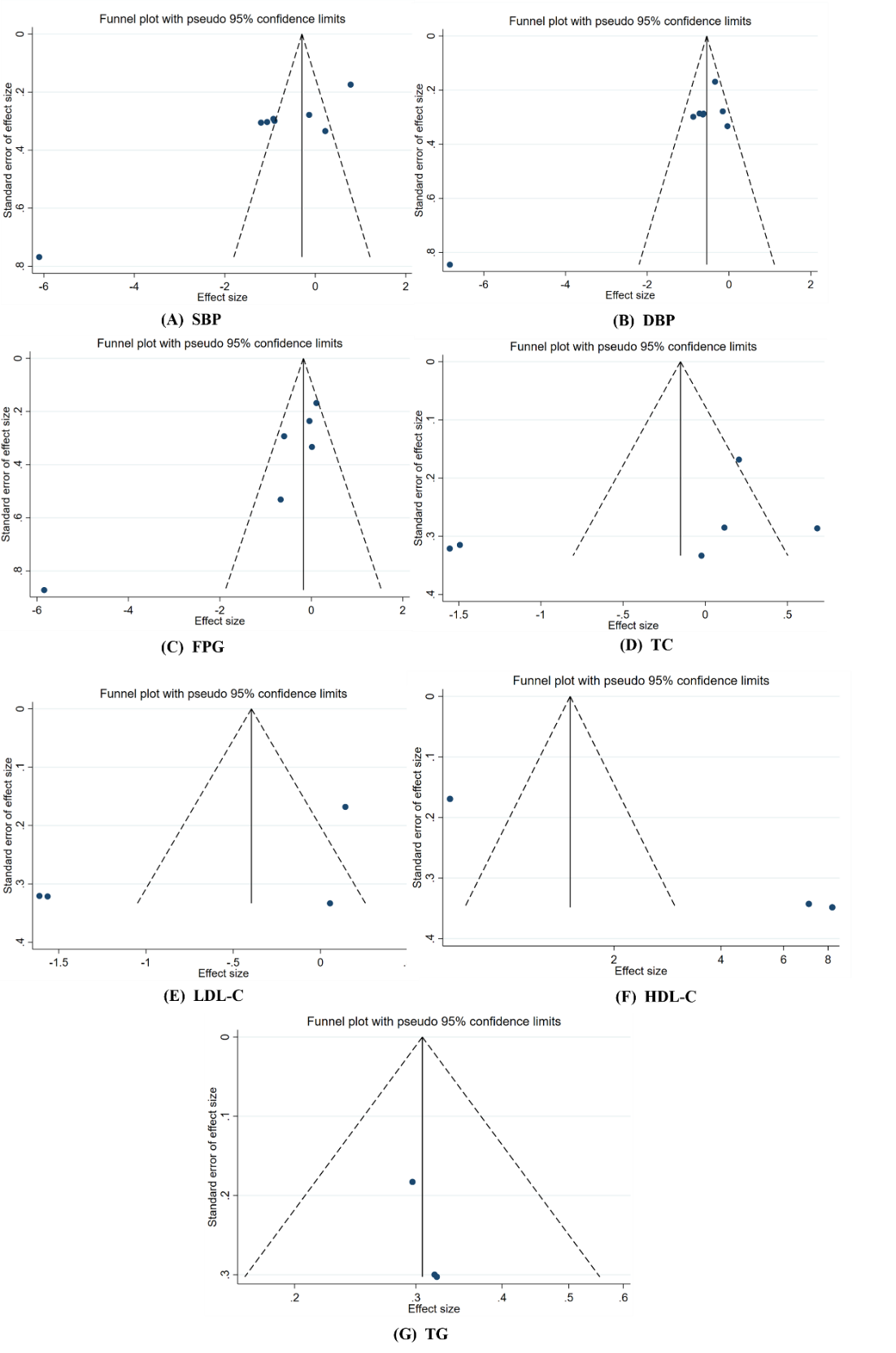


**Figure S4.1.** Funnel plot representing publication bias for the impact of berberine intake in (A) systolic blood pressure (SBP); (B) diastolic blood pressure (DBP); (C) fasting plasma glucose (FPG); (D) total cholesterol (TC); (E) low-density lipoprotein cholesterol (LDL-c); (F) high-density lipoprotein cholesterol (HDL-c); (G) triglycerides (TG)


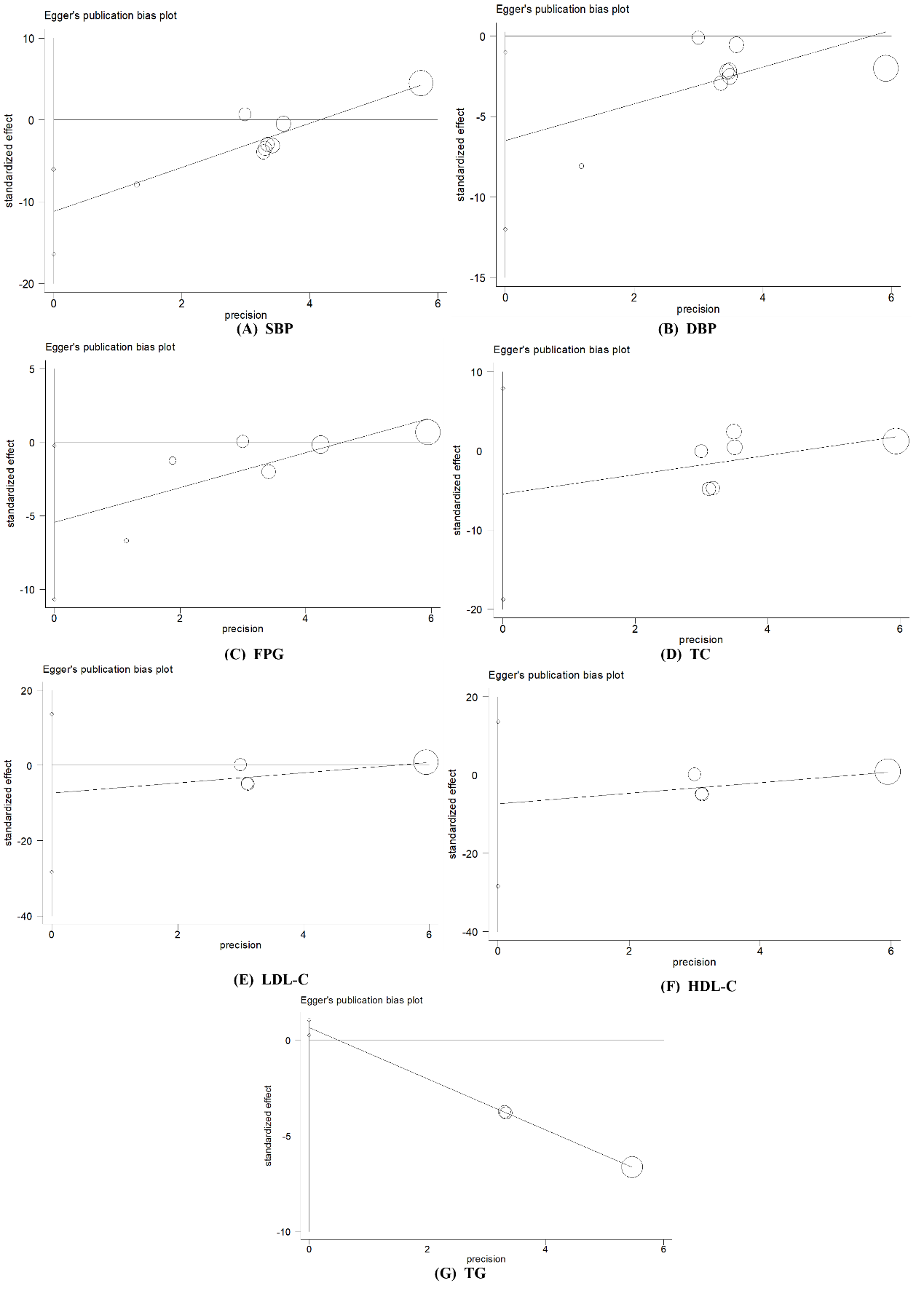


**Figure S4.2.** Egger's test for assessing publication bias. (A) SBP Egger's test (*p* = 0.004); (B) DBP Egger's test (*p* = 0.114); (C) FPG Egger's test (*p* = 0.085); (D) TC Egger's test (*p* = 0.385); (E) LDL-C Egger's test (*p* = 0.382); (F) HDL-C Egger's test (*p* = 0.015); (G)TG Egger's test (*p* = 0.004).


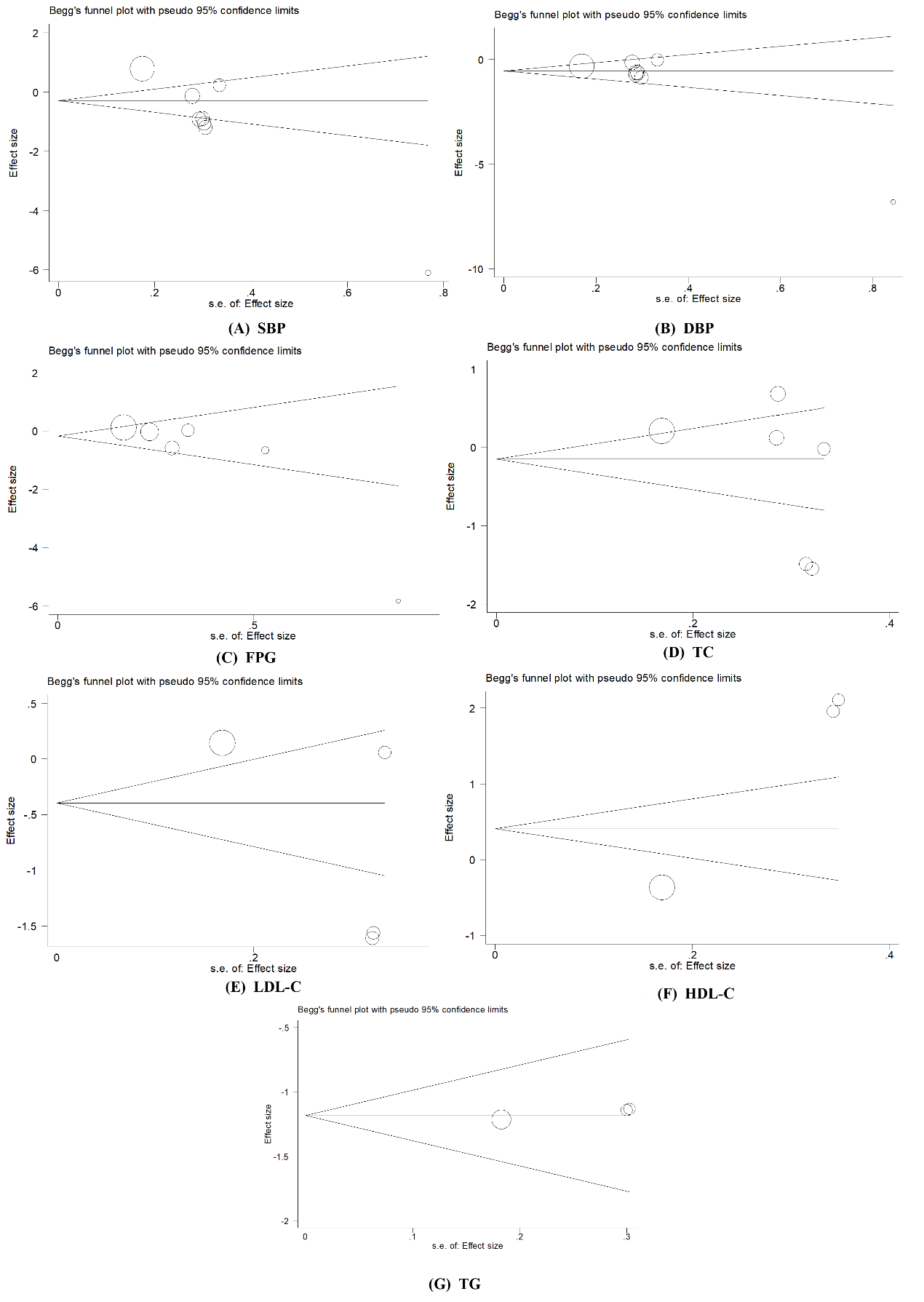


**Figure S4.3.** Begg's test for assessing publication bias. (A) SBP Begg's test (*p* = 0.063); (B) DBP Begg's test (*p* = 0.266); (C) FPG Begg's test (*p* = 0.060); (D) TC Begg's test (*p* = 0.260); (E) LDL-C Begg's test (*p* = 1.000); (F) HDL-C Begg's test (*p* = 0.296); (G)TG Begg's test (*p* = 0.296).

**
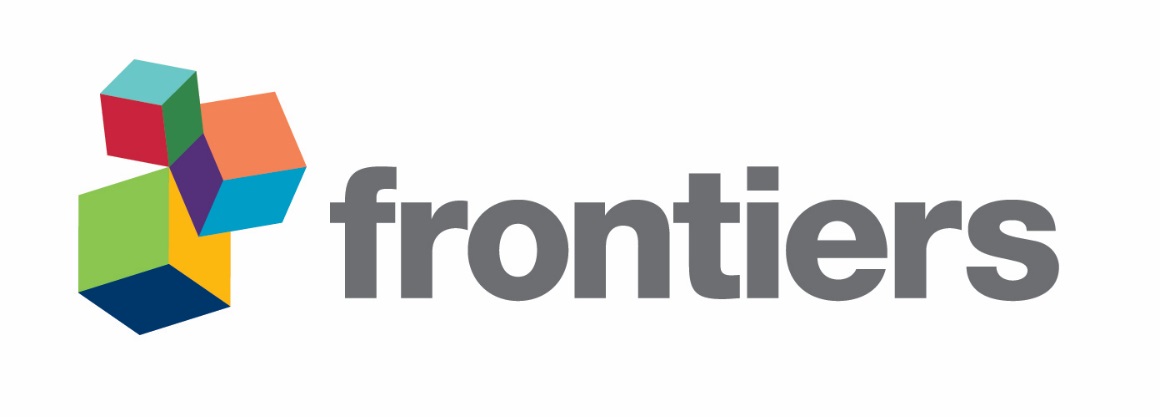
**

# 5 References

(2012). Methods for guideline development. *Kidney Int Suppl (2011)* 2(5)**,** 388-397. doi: 10.1038/kisup.2012.59.

(2024). "NICE Evidence Reviews Collection," in *Evidence reviews for stratifying risk of severe illness or death from sepsis: Suspected sepsis: recognition, diagnosis and early management: Evidence review A*. (London: National Institute for Health and Care Excellence (NICE)

Copyright © NICE 2024.).

Ettehad, D., Emdin, C.A., Kiran, A., Anderson, S.G., Callender, T., Emberson, J., et al. (2016). Blood pressure lowering for prevention of cardiovascular disease and death: a systematic review and meta-analysis. *Lancet* 387(10022)**,** 957-967. doi: 10.1016/s0140-6736(15)01225-8.

Goldkuhle, M., Guyatt, G.H., Kreuzberger, N., Akl, E.A., Dahm, P., van Dalen, E.C., et al. (2023). GRADE concept 4: rating the certainty of evidence when study interventions or comparators differ from PICO targets. *J Clin Epidemiol* 159**,** 40-48. doi: 10.1016/j.jclinepi.2023.04.018.

Guyatt, G., Oxman, A.D., Kunz, R., Brozek, J., Alonso-Coello, P., Rind, D., et al. (2021). Corrigendum to GRADE guidelines 6. Rating the quality of evidence-imprecision. J Clin Epidemiol 2011;64:1283-1293. *J Clin Epidemiol* 137**,** 265. doi: 10.1016/j.jclinepi.2021.04.014.

Kelu, Y., Long, G., Ming, L., Ya, G., Liwei, S., Yue, X., et al. (2020). Application of GRADE in health technology assessment. *Chinese Journal of Drug Evaluation* 37(06)**,** 411-416.

Saueressig, T., Braun, T., Steglich, N., Diemer, F., Zebisch, J., Herbst, M., et al. (2022). Primary surgery versus primary rehabilitation for treating anterior cruciate ligament injuries: a living systematic review and meta-analysis. *Br J Sports Med* 56(21)**,** 1241-1251. doi: 10.1136/bjsports-2021-105359.
